# Sparse Coding Can Predict Primary Visual Cortex Receptive Field Changes Induced by Abnormal Visual Input

**DOI:** 10.1371/journal.pcbi.1003005

**Published:** 2013-05-09

**Authors:** Jonathan J. Hunt, Peter Dayan, Geoffrey J. Goodhill

**Affiliations:** 1 Queensland Brain Institute, University of Queensland, St Lucia, Australia; 2 Gatsby Computational Neuroscience Unit, University College London, London, United Kingdom; 3 School of Mathematics and Physics, University of Queensland, St Lucia, Australia; University of Tuebingen and Max Planck Institute for Biologial Cybernetics Tuebingen, Germany

## Abstract

Receptive fields acquired through unsupervised learning of sparse representations of natural scenes have similar properties to primary visual cortex (V1) simple cell receptive fields. However, what drives *in vivo* development of receptive fields remains controversial. The strongest evidence for the importance of sensory experience in visual development comes from receptive field changes in animals reared with abnormal visual input. However, most sparse coding accounts have considered only normal visual input and the development of monocular receptive fields. Here, we applied three sparse coding models to binocular receptive field development across six abnormal rearing conditions. In every condition, the changes in receptive field properties previously observed experimentally were matched to a similar and highly faithful degree by all the models, suggesting that early sensory development can indeed be understood in terms of an impetus towards sparsity. As previously predicted in the literature, we found that asymmetries in inter-ocular correlation across orientations lead to orientation-specific binocular receptive fields. Finally we used our models to design a novel stimulus that, if present during rearing, is predicted by the sparsity principle to lead robustly to radically abnormal receptive fields.

## Author Summary

The responses of neurons in the primary visual cortex (V1), a region of the brain involved in encoding visual input, are modified by the visual experience of the animal during development. For example, most neurons in animals reared viewing stripes of a particular orientation only respond to the orientation that the animal experienced. The responses of V1 cells in normal animals are similar to responses that simple optimisation algorithms can learn when trained on images. However, whether the similarity between these algorithms and V1 responses is merely coincidental has been unclear. Here, we used the results of a number of experiments where animals were reared with modified visual experience to test the explanatory power of three related optimisation algorithms. We did this by filtering the images for the algorithms in ways that mimicked the visual experience of the animals. This allowed us to show that the changes in V1 responses in experiment were consistent with the algorithms. This is evidence that the precepts of the algorithms, notably sparsity, can be used to understand the development of V1 responses. Further, we used our model to propose a novel rearing condition which we expect to have a dramatic effect on development.

## Introduction

Simple cells in the mammalian primary visual cortex (V1) are among the cells in the brain that are best functionally characterised [1]–[3]. They have also been used as a key model system for studying the complex interplay of intrinsic and extrinsic factors, i.e., nature and nurture, in controlling development. For instance, there is ample evidence that receptive field structure exists prior to eye-opening [e.g. 4]–[6], being significantly present in dark-reared animals [7], [8]. Yet numerous studies, many taking advantage of the fact that simple cells are the earliest in the visual pathway to encode input from both eyes [9], have demonstrated that receptive field properties are modified by visual experience during development [e.g. 10]–[20].

Developing a general theory of sensory coding has been an important goal of computational neuroscience. One famously powerful idea, Barlow's efficient coding hypothesis, is that early sensory coding attempts to remove redundancy by representing input in informationally optimal ways [21]. Among other achievements, this hypothesis has provided compelling explanations for the characteristics of retinal receptive fields [22]. However, redundancy reduction may be only a first step in sensory processing [23]. For instance, V1 is many times overcomplete in its representation of input [24], a fact that, on the surface at least, increases rather than decreases the redundancy in the encoding of the input [25].

One possibility is that V1 is attempting to code visual input sparsely [26]. Many variants of sparse coding have been mooted [27]–[35], and, when tailored for natural scene input, almost ubiquitously lead to units with response properties similar to V1 simple cells. Other work has extended sparse coding models of V1 to complex cells [36], the dimension of time [37] and color [38], [39] [reviewed in 33]. Sparse learning schemes often trade off the amount of sparsity and the error in the encoding. The justification for sparse coding has ranged from the energetic grounds of being metabolically efficient [40], to the statistical grounds of exposing underlying latent structure in the input [24], [31].

The boldest claim of the sparse coding hypothesis is that it offers more than just an interpretation of simple cell receptive fields, but rather that it can account for the outcome (if not necessarily the time-course) of cortical plasticity. Showing this would offer a more stringent response to criticisms about the utility of these forms of unsupervised learning models for understanding visual development [20], [41], and also license applications of the same principles at more advanced stages of sensory processing. However, bar some notable exceptions [e.g. 35], [42], models based on precepts such as sparse coding have typically been applied to the development under normal input, for which the role of nurture can be questioned, rather than under abnormal input, for which it cannot. Furthermore, apart from notable exceptions such as Hoyer and Hyvärinen [39], the models have typically focused on monocular rather than binocular receptive fields, thus not addressing many of the most important experimental conditions.

Here we tested whether receptive field changes in six abnormal rearing conditions applied to cats (table 1) can be captured by binocular versions of sparse coding models of receptive field development. The cat was chosen as the model organism to match since all the conditions have been examined experimentally by several different groups, leading to broad agreement in the results. The more limited range of experiments that have been conducted in other species, notably macaques, have led to similar results, as in [43].

**Table 1:** Rearing conditions.

Rearing	Description of visual input	Salient changes in receptive fields	References
Normal	Normal visual input.	n/a	[1], [153], [154]
Stripe	Animals were exposed to a single orientation in both eyes using goggles or a striped environment.	Over-representation of the reared orientation. A reduced number of neurons were strongly orientation selective. Increased binocularity.	[15], [49]–[57]
Orthogonal	Animals were exposed to horizontal orientations in one eye and vertical orientations in the other eye using goggles.	Increased monocularity. Reduced number of neurons with well-defined orientation preferences. Over-representation of the reared orientation in each eye.	[52],[54],[55],[58]–[61]
Monocular	Animals were reared with one eye occluded.	Majority of neurons were responsive to the non-occluded eye. A small minority were responsive to the occluded eye, almost all neurons were extremely monocular.	[12]–[14], [16], [55], [62]–[73], [76], [78]
Alternating-monocular	Animals were reared with only one eye open at any time, but the occluded eye was regularly alternated.	Strongly monocular receptive fields but with equal representation of both eyes and all orientations.	[10], [55], [74]–[76], [79]–[81], [84]
Partial-monocular	Animals were reared with one eye occluded but were given a small amount of binocular experience.	Recovery of near-equal representation of both eyes, but with few binocular responses and poor depth perception.	[16]–[19], [75], [78], [82]–[84]
Strabismic	Nonparallel visual axis (achieved artificially by severing extra-ocular muscles or with prisms).	Normal orientation coverage but with few binocular responses	[10], [12], [18], [55], [74], [87], [89]–[96]

We modelled receptive field development in normal and six abnormal rearing conditions. This table provides a summary of the receptive field changes observed in each condition along with references to the original experiments. In our model, abnormal conditions were simulated by filtering the binocular training input to be consistent with the visual experience of the abnormally reared animals.

To ensure the outcomes were due to the general principle of sparsity, rather than the specifics of a particular algorithm, we used three different generative models for learning sparsity: product of experts [44], k-means clustering and independent component analysis [45]. We found that all three models qualitatively reproduced the receptive field changes observed in experiment in every rearing condition considered, and provided a good quantitative match in cases in which there was sufficiently ample sampling of receptive fields in the relevant experiments. This agreement provides evidence that receptive fields are indeed optimized during development in response to input statistics. Further, we used our models to design a novel rearing condition that we propose offers a strong test of the explanatory power of sparse coding. This involves presenting white noise with sparsity greater than that of natural scenes such that, even when augmented with natural input, it is still expected to lead to the development of highly localized receptive fields that are quite different from those of normal simple cells.

Overall, we suggest that examining abnormal rearing conditions will offer tests of functional accounts of development that are revealing and stringent, and look forward to the prospect of their application to higher visual areas and other sensory modalities.

## Results

We examined whether simple unsupervised learning models could capture the receptive field structures observed in abnormally reared animals. The models learn sparse responses which are conditionally independent given the input. We considered normal rearing, along with six abnormal rearing conditions (summarised in table 1).

**Figure 1:**
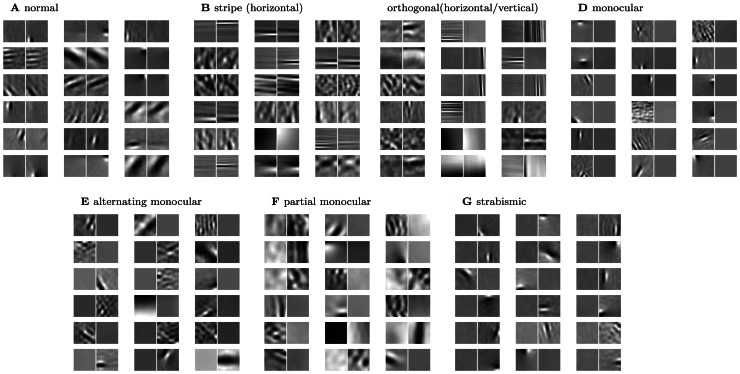
Example receptive fields (PoE model). Representative examples of the V1 receptive fields over both eyes that result for the PoE model (lower 18 pairs). Subsequent figures quantify the changes in receptive field structure and distribution induced in each rearing condition. See table 1 for a summary of the receptive field changes seen experimentally for each condition. We model rearing with (**A**) normal (unfiltered) visual input, (**B**) stripe rearing, i.e. a single dominant orientation (in this case horizontal), (**C**) orthogonal stripe rearing, i.e. dominant orientations differing by 90 degrees between the two eyes (in this case horizontal and vertical), (**D**) monocular deprivation, i.e. one eye occluded, (**E**) one eye occluded but alternating the eye randomly during training, (**F**) one eye occluded most of the time, and (**G**) artificial strabismus (direction of gaze offset between the two eyes).

All three unsupervised learning models gave qualitatively similar results across the rearing conditions. Figures shown in the main text, starting from the bottom rows in each column in Figure 1 which show sample receptive fields for each rearing condition, are for the results found using the product of experts model [44]. In Text S1/S2 we provide the same figures with the results for independent components analysis/k-means clustering. Where there are notable differences between the models we mention this is the main text.

**Figure 2:**
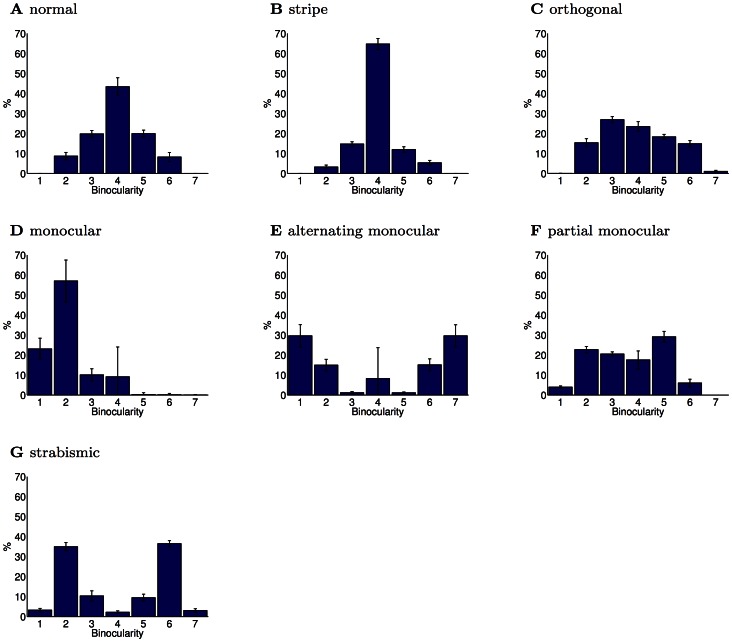
Degree of binocularity for rearing conditions (PoE model). Binocularity was measured on a 7 point scale as in Shouval et al. [150]. Values 1 and 7 represent completely monocular responses while values in the middle correspond to at least somewhat binocular responses. (**A**) In the normal rearing condition, neurons had a range of binocular responses, although there were few completely monocular neurons. (**B**) In the stripe-reared condition, binocularity increased due to higher inter-ocular correlation caused by the reduction in off-axis spatial frequencies. Experiments have also reported varying amounts of increases in binocular responses. (**C**) In the orthogonal-reared condition, binocularity decreased. Experiments have also reported such a decrease. (**D**) In the monocular-reared condition, neurons developed responses primarily for the unoccluded eye, which led to strongly monocular responses for this eye. The primary experimental finding in this rearing condition has been the absence of responses to the occluded eye. (**E**) Alternating monocular rearing removes inter-ocular correlation as each eye is presented with stimuli only when the other eye is occluded. In the PoE model, this led to strongly monocular responses distributed equally between eyes. Experimentally, the primary finding has been a paucity of binocular responses, but equal responses to each eye. (**F**) Partial monocular rearing resulted in recovery of receptive fields for both eyes, albeit with fewer binocular neurons. Experimentally, a small amount of binocular experience has been found to result in a significant recovery of responses to the occluded eye, but also an increased degree of monocularity. (**G**) Strabismus decreases inter-ocular correlation, and thus led in the PoE model to increased monocularity. An increase in monocularity is the primary experimental finding of the effects of strabismus. Errorbars show the SEM. Each condition was repeated 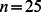 times. The binocularity distribution of all the modified rearing conditions were significantly different from the normal rearing condition (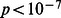, Kolmogorov-Smirnov).

**Figure 3:**
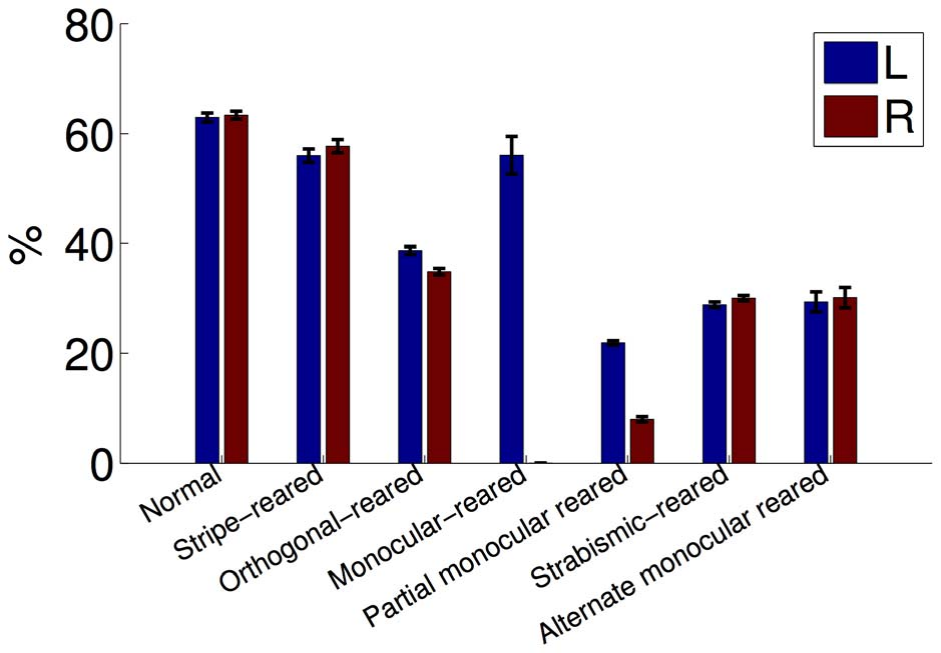
Orientation selectivity across rearing conditions (PoE model). As in experiment, filtering the visual input resulted in a decrease in the fraction of orientation selective neurons (each eye shown separately). Neurons were considered selective when their circular variance was 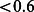 [3]. Errorbars show the SEM. All modified rearing conditions had a significantly different fraction of orien- tation selective neurons compared with the normal condition (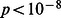, Kolmogorov-Smirnov, each eye tested separately).

**Figure 4:**
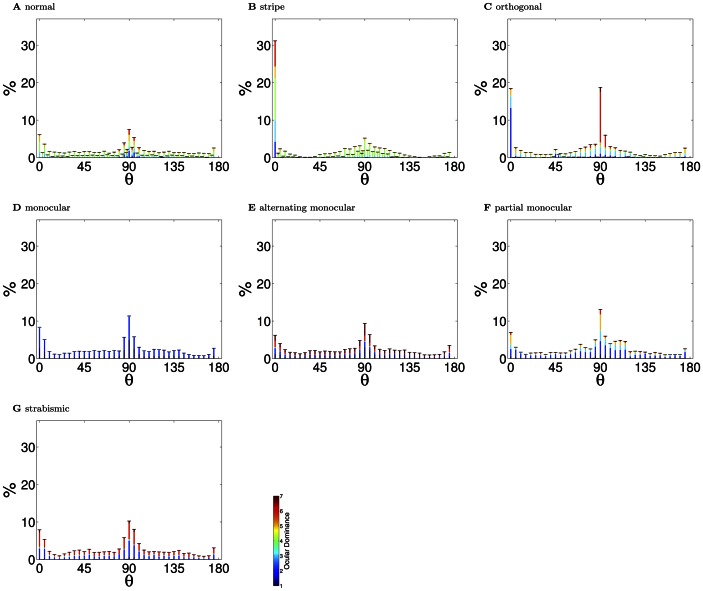
Orientation preference distributions (PoE model). Only neurons that had a well defined orientation preference (circular variance 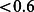) in at least one eye were included. The color in the bars indicates the ocular dominance of the responses. (**A**) In the normal reared condition, there was a large over-representation of vertical orientations (90°), and, to a lesser degree, horizontal orientations (0°). Nonetheless, the full range of orientation preferences developed. This over-representation of the cardinal orientations has been reported in experiment, although not always to the same degree (see the Discussion). (**B**) In the stripe reared condition, there was a significant over-representation of neurons responding to horizontal lines (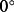). These horizontal neurons were also strongly binocular (i.e. mostly green shading). The over-representation of vertical orientations also persisted. The over-representation of the reared orientation is the primary experimental finding in this rearing condition. Cardinal over-representation has not been examined closely in stripe-rearing. (**C**) In the orthogonally reared condition, there was over-representation of horizontal neurons in the left eye and vertical neurons in the right eye. As found experimentally, these neurons were strongly monocular for the eye that was over-exposed to their preferred orientation. (**D**) In the monocular reared condition, there was a broad representation of orientation preferences but only for the unoccluded eye. Experimentally, monocular reared animals have normal visual acuity with the non-deprived eye. (**E**) In the alternating monocular reared case, there is an even distribution of orientation selectivity and strong monocularity. Experimentally, alternate blind reared animals represent all orientations well. (**F**) In the partial monocular reared condition, there was a recovery of responsivity for both eyes across the full range of orientations. Experimentally, partial monocular reared animals have been demonstrated to have normal visual in each eye (but to suffer from defects in stereo vision). (**G**) In the strabismic case, there was an increase in monocularity, but with normal orientation coverage. This is in agreement with experiments which have not noted any orientation deficits in strabismic animals. Errorbars show the SEM. All modified rearing cases, except strabismus (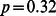) had orientation preference distributions significantly different from the normal case (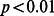, Kolmogorov-Smirnov).

To facilitate a direct comparison between models and experiment, in each of the subsequent sections we first provide a brief literature review of the relevant experimental work for that condition, and then present the results of our models. Although some conditions required specialised additional comparisons, changes were observed in every condition in receptive field binocularities (figure 2), the fraction of oriented receptive fields devoted to each eye (figure 3) and the joint orientation-binocularity distribution (figure 4). These figures are referred to in each section. In sum, all three models are able to match the changes in receptive field properties observed across all the conditions considered.

### Normal rearing (figures 1A, 2A, 4A)

As expected from previous work in the monocular case [27], [28], [31], [32], the binocular receptive fields learned based on normal input were Gabor-like edge detectors (figure 1**A**). This property broadly survived the modified rearing conditions, up to some degradation and broadening. Given normal input, receptive fields were distributed over the full range of orientations (figure 4**A**) with primarily binocular responses (figure 2**A**). Note that the quantification of binocularity in some early experimental results is somewhat subjective, which complicates quantitative comparison. For example, most experimental groups classify monocular cells as ones which respond solely to one eye, which is difficult to define theoretically, since learned receptive fields are never entirely empty.

One salient feature of the orientation distribution is the over-representation of cardinal orientations. There is evidence that some degree of cardinal over-representation is present in normally reared animals [46] and in the visual environment [47], although it may be accentuated in our work due to the pixel representation of the training images (see later for further discussion and references).

An additional feature of note is the relationship observed in the normal rearing condition between orientation and binocularity. Li and Atick [48] examined 2nd-order correlations in visual input and predicted that vertically oriented receptive fields should be more monocular than horizontally oriented ones due to the asymmetry in inter-ocular correlations with horizontal disparity. This asymmetry in encoding can be seen in the normal case (figure 4**A**), with significantly more monocular receptive fields for vertical orientations. We are not aware of detailed experimental investigation of this phenomena (see Discussion).

**Figure 5:**
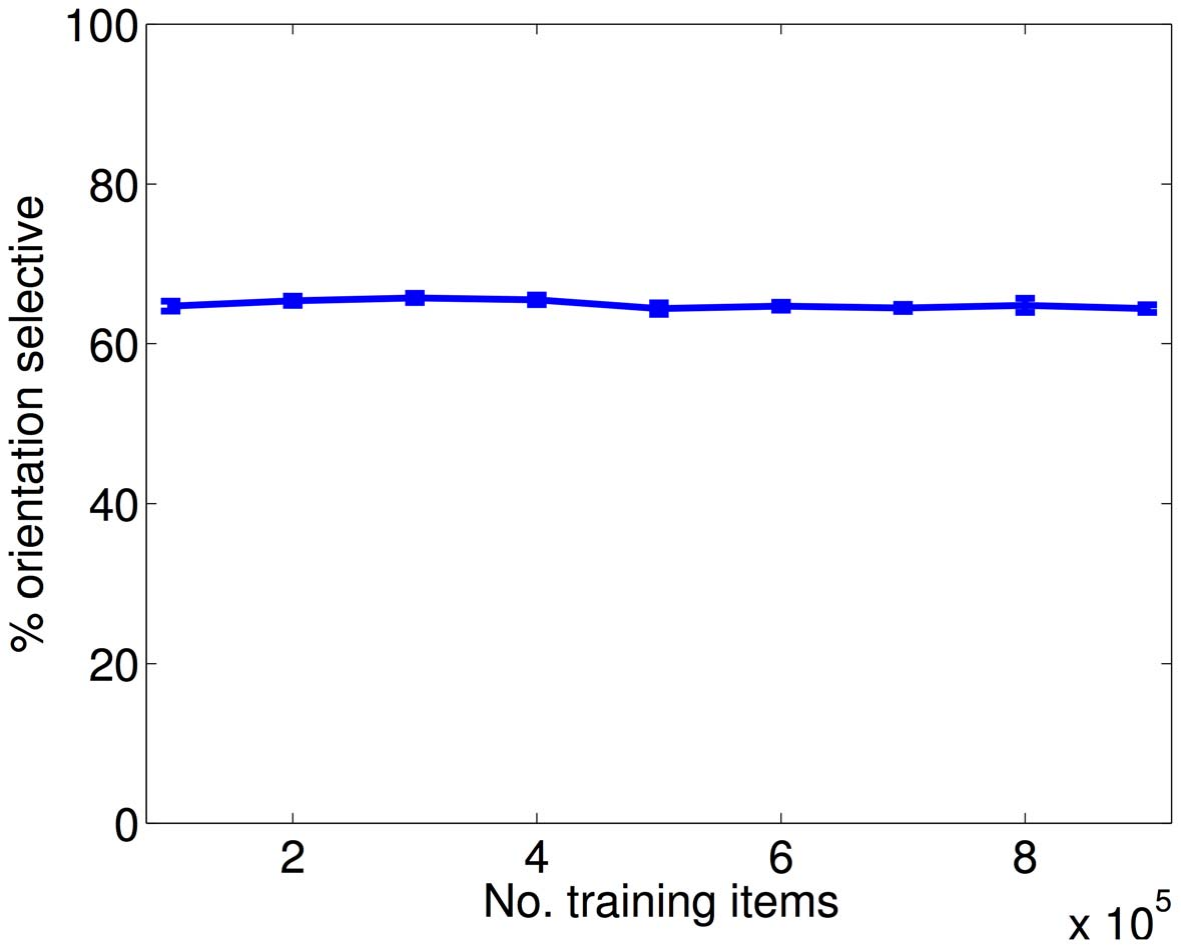
Model is robust to training set size (PoE model). The PoE model has approximately 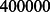 degrees of freedom. However, for computational reasons, we trained the model with only 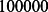 training examples (each training example is projected onto 150 principal components). We therefore examined the effect of increasing the training set size (for the case of normal input). The receptive field orientation selectivity was robust to changes in training set size. There was also no obvious visual change in receptive field structure (data not shown). This demonstrates that the sparsity constraints result in receptive field formation robust to training set size.

One concern we examined for the case of normal input was the robustness of the models to training set size. This is particularly important since, for computational reasons, we trained our models with 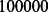 training examples, which is approximately a factor of four less than the number of degrees of freedom of the overcomplete models. By inspection, receptive fields appeared similar for different training set sizes. To test this more quantitatively, we examined the dependence on size of a key statistic of the receptive fields, namely orientation selectivity (figure 5). We found no dependence. Thus, the sparse constraints of the model result in a fit that is robust to training set size, even in the under-constrained regime.

### Stripe rearing (figures 1B, 2B, 4B, 6)

Stripe rearing refers to the condition in which animals are raised with visual experience consisting primarily of a single orientation. This can be achieved by the use of cylindrical lenses, lenses painted with stripes, or rearing chambers with striped walls. Early electrophysiological studies on the effects of stripe rearing conflict, with some reports of a complete absence of receptive fields responsive to the unexperienced orientations [49], [50], while others found no effect on receptive field distribution [51], and Freeman and Pettigrew [52] found a more limited over-representation of the experienced orientation and reduced selectivity to the unexperienced orientations. Later experiments found significant over-representation of the experienced orientation [53], [54] and reduced orientation selectivity for unexperienced orientations. Unlike the other studies, Freeman and Pettigrew [52] and Blasdel et al. [53] reported a reduction in binocular responses; however Freeman and Pettigrew [52] attributed this to misalignment in the oriented lines between the two eyes. Blakemore [55] found that stripe reared animals have normal levels of binocularity.

More recently, optical imaging techniques have allowed simultaneous characterisation of large regions of V1. Using optical imaging, Sengpiel, Stawinski and Bonhoeffer [15] found a roughly 60% increase in the cortical area devoted to the over-represented orientation, with no change in the orientation selectivity between the experienced and unexperienced orientations. Tanaka et al. [56], [57] used exclusive goggle rearing with cylindrical lenses and found a much more dramatic 3–6 fold over-representation and increased selectivity (and reduced variance) of the exposed orientation. Tanaka and colleagues also noted that older animals exhibited reduced over-representation despite continued goggle rearing, and that increased dark exposure limited the effect of the goggle rearing.

There are several possible explanations for some of the differences between studies. The method of over-representation varied: some studies used stripe-cylinders, while others used goggles containing lines or strong cylindrical lenses. The age and duration of exposure also varied, and some early studies may have suffered from sampling biases. However, there is broad agreement between studies that stripe rearing leads to increased binocularity and significant increases in over-representation of the exposed orientation. Further, several studies found increased selectivity of the exposed orientation.

**Figure 6:**
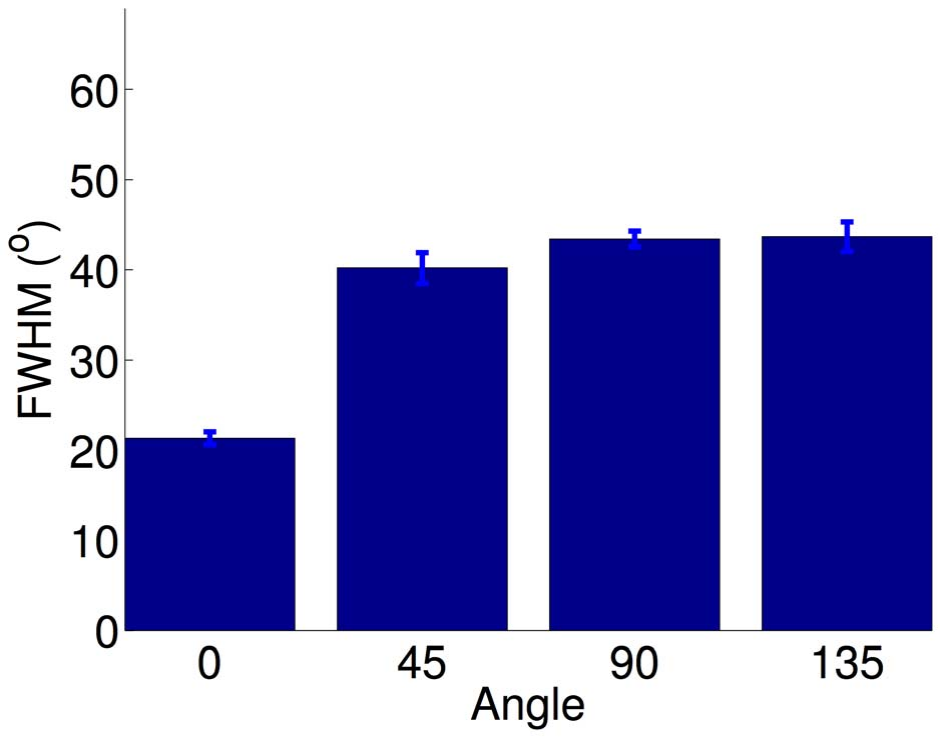
Tuning width in stripe-rearing (PoE model). In the PoE model, horizontal stripe rearing resulted in significantly sharper orientation tuning for the over-exposed orientation (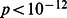 Kruskal-Wallis). Experimentally, there are conflicting results regarding the tuning of neurons representing the exposed orientation. Tuning width was measured as the full width at half maximum (FWHM) of the tuning curve.

We modeled stripe-rearing by filtering the input using oriented Gaussian blurring designed to attenuate the power of off-axis orientations sharply. To maintain stability of the algorithm, 10% normal images were included in the training mixture (as in [42], see Discussion). The output of the models was consistent with the experimental observations. Receptive fields trained on striped input showed increased binocularity (figure 2**B**, 8% monocular cells in the stripe-reared condition compared with 17% monocular cells in normal condition, 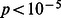 two-sided t-test). As in the experiments, there was a slight reduction in the number of orientation selective responses (figure 3), and the over-exposed orientation (in our case, horizontal) had sharper tuning curves (figure 6) with a smaller variance in their tuning. The size of these changes was dependent on the strength of the input filtering (data not shown), which is another possible explanation for the differing effect sizes seen between groups using different rearing techniques. These changes collectively meant that many receptive fields were less Gabor-like, although orientation selectivity was largely preserved. Since we could not find empirical studies employing methods such as reverse correlation against which to compare our results, it is difficult to determine how faithful this result is. However, the loss of structure of some receptive fields is at least qualitatively consistent with the experimental finding of reduced orientation selectivity in stripe rearing.

### Orthogonal rearing (figures 1C, 2C, 4C)

Orthogonal rearing is a binocular extension of stripe rearing in which the two eyes are exposed to orthogonal orientations. Hirsch and Spinelli [58], [59] found that orthogonally reared animals had reduced binocularity and almost exclusively monocular responses for cells with well-defined orientations. They also found an almost perfect correlation between receptive field ocular and orientation preferences along with an overall reduction in the fraction of oriented responses. Subsequent groups found similar results although the quantitative changes reported varied, possibly due again to differences in the type of filtering and the strength of the manipulation between experiments. Freeman and Pettigrew [52] noticed reduced binocularity (25–35% binocular cells for orthogonally reared animals compared with 85% for normally reared animals) and a strong correlation between orientation and ocular preference along with broader orientation tuning away from the over-exposed orientations. Blakemore [55] also noted reduced binocularity (53% monocular responses).

Leventhal and Hirsch [60] observed a difference between horizontal and vertical orthogonal rearing and orthogonal rearing with oblique angles. In the cardinal case, they found a strong correlation between ocular and orientation preference. With oblique angles they found a continued dominance of horizontal and vertical orientations, but little response to the non-exposed non-cardinal orientation. In all cases they noted a reduction in binocularity. Stryker et al. [54] also found a reduction in the number of oriented responses (50% of cells were not responsive or not selective to orientation) and strong correlation between eye and orientation preference. They also observed over-representations of approximately two-fold for the exposed orientations and almost no binocular cells. More recently, albeit as yet only in abstract form, Tani and Tanaka [61] confirmed the over-representation of the exposed orientations using optical imaging.

As in the stripe reared case, we modelled orthogonal rearing by oriented Gaussian blurring. However, in this condition, the left eye viewed horizontally filtered images while the right eye viewed vertically filtered images. This led to similar results as in the experiments. When trained on this orthogonally filtered input, model receptive fields were significantly more monocular (figure 2**C**, 31% monocular compared with 17% in the normal case, 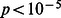, two-sided paired ttest), although this effect was not as pronounced as reported experimentally. Responses showed a strong correlation between ocular and orientation preference (figure 4**C**). Additionally, there was a reduced fraction of oriented responses compared with the normal case (figure 3, 37% compared with 63% in the normal case, 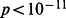). The results were similar when oblique rearing orientations are considered, although in this case there was also a smaller, cardinal over-representation effect (data not shown). This cardinal over-representation is presumably driven by the same causes as in normal case (discussed further below): cardinal over-representation in the input and the square pixel representation.

### Monocular rearing (figures 1D, 2D, 4D)

Monocular deprivation, in which one eye is deprived of visual input, is perhaps the best-studied manipulation. There is substantial variation in deprivation length and daily visual exposure between different studies. We only considered results based on experiments with no recovery period with normal visual input. We examine the recovery of binocular fields later in the section on partial monocular rearing.

Early work by Wiesel and Hubel [62], [63] using electrophysiology found almost no response to the deprived eye (1/84 cells responded). Similarly, Hubel and Wiesel [64] observed only 7% of cells responsive to the deprived eye after 3 months of deprivation (all cells were classified as having ocular dominance values of 6 or 7 on a scale of 0–7). Blakemore and Van Sluyters [65] also demonstrated almost complete domination of V1 by the deprived eye, with normal levels of orientation selectivity in the non-deprived eye. Using autoradiography, Shatz, Lindstrom and Wiesel [12], Stryker [14] and Shatz and Stryker [13] found shrinkage of the deprived eye's territory with only 22–25% cortical area labelled by the deprived eye. Olson and Freeman [66] considered the effects of shorter periods of deprivation, finding pronounced decreases in binocularity after just 2.5 days of deprivation, and almost total loss of responsiveness to the deprived eye after longer periods, compared with 80% binocular responses in the normal animals. Similarly, Peck and Blakemore [67] found that, with just 20 hours of monocular deprivation, all oriented cells had ocular dominances in the range 4–7. Only a small number of unoriented responses remained exclusive to the deprived eye. Schechter and Murphy [68] noted 86% of cells responded exclusively to the open eye, 3% to the deprived eye, 3% had binocular responses and 7% were unresponsive. Kratz and Spear [69] observed a reduced number of orientation selective cells (65% compared with 85% in normal) and reduced direction selectivity (70% compared with 90% in normal). Blasdel and Pettigrew [70] found that 3 weeks of molecular deprivation led to most cells having ocular dominances of 7, with a small number reported as being 5 and 6. Olson and Freeman [71] also noted that only 3% of cells responded to the deprived eye (87% in normal) after 10 days of monocular deprivation. Singer et al. [72] found that the majority of cells were responsive only to the open eye.

Some variations have added insight about the changes occurring during monocular deprivation. Blakemore [55] and Wilson, Webb and Sherman [73] demonstrated that there was little difference between monocular deprivation with the nictitating membrane or the full eye-lids, showing that the loss of spatial patterns, rather than the change in luminance, is the critical component of deprivation. Blakemore and Hillman [74] showed that the open eye dominated whether optically stimulated, or driven electrically. Olson and Freeman [75] interspersed dark-reared intervals during the deprivation and continued to find almost no response to the deprived eye. Tumosa, Tieman and Hirsch [76] used behavioral assays to show that the animals had no functional visual acuity in the deprived eye. Mitchell [77] demonstrated that recovery was improved when the non-deprived eye was occluded during the recovery period. More recent experiments have used optical imaging, and found only 14–18% of cortex responded to the occluded eye [16] and confirmed that little functional visual acuity remains in the deprived eye [78].

We used the observation from Wilson, Webb and Sherman [73] that it is the spatial pattern of the input associated with the deprived eye that matters rather than the overall power to realize a stringent test of the model. We simulated this by using an extremely low-pass boxcar filter on the right eye's input, so that almost all contrast was destroyed. The model reproduced the experimental findings, producing primarily cells with ocular dominance values of 2, and none greater than 4 (figure 2**D**), indicating no cell responded more strongly to the deprived eye than the open eye. No oriented response was assigned to the deprived eye (figure 3).

### Alternating monocular rearing (figures 1E, 2E, 4E)

In alternating monocular rearing, animals are monocularly deprived, but which eye is deprived is alternated regularly (every few hours of visual experience). This removes all inter-ocular correlations, while not favouring the development of either eye. Hubel and Wiesel [10] first performed this experiment and found that 91% of the resulting cells had monocular responses, evenly distributed between the two eyes. Behaviourally, the animals appeared to have normal spatial acuity in each eye. Blake and Hirsch [79] also measured normal acuity in each eye but observed defects in stereopsis. They also noted an almost complete absence of binocular responses even after animals were reared with normal input for a year after the critical period. Blakemore [55] found alternating monocular rearing resulted in 55% of neurons responding monocularly, similar to a strabismic animal. Blasdel and Pettigrew [80] also found reduced binocularity (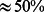 binocularity) except when they used a mechanised device to reduce the alternation interval to less than 1 second. They also observed a low correlation in orientation tuning between the two eyes (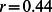 versus 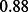 in normal animals). Tumosa, Tieman and Hirsch [76] used behavioural assays and found normal visual acuity in each eye, and equal cortical coverage devoted to each eye [81]. In all of these experiments, alternating monocular rearing had a similar effect to strabismic rearing: reduced binocularity while retaining an equal number of neurons devoted to each eye.

We simulated this condition in a similar way to monocular rearing, with the blind eye having its input low-pass filtered so that little contrast remained. The difference from monocular rearing was that the eye that was blind was alternated for each patch. This resulted in quantitative agreement with experiment. The PoE model predicted (figure 2**E**) 89% monocular responses (compared with 17% in the normal case, 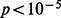, two-sided paired t-test) and a symmetrical ocular dominance distribution. There was also a reduced fraction of oriented responses (figure 3).

### Partial-monocular rearing (figures 1F, 7, 8)

Monocular rearing leads to almost complete loss of function in the deprived eye. There has thus been substantial interest in the question as to what features of the input are necessary for preventing this. Partial-monocular rearing, in which animals are exposed to a small fraction of binocular experience, allows the amount of normal input needed for the maintenance of responses to both eyes to be determined.

Olson and Freeman [75] monocularly deprived kittens for 4 hours while providing 14–20 hours of binocular experience per day. They found this limited deprivation had little effect. Similarly, Kind et al. [16] examined the effect of monocular deprivation for 10 days (at 5 weeks old) followed by binocular exposure for 14 days. Again, these kittens had nearly normal visual development, although non-aligned binocular input (i.e. strabismic) led to only 34% coverage for the deprived eye, demonstrating that correlated visual input may be important for recovery. Conversely, Malach and Van Sluyters [82] found that strabismic binocular input did lead to recovery of binocular responses, but this may be because the animals were dark-reared for 18 hours per day, a manipulation that is known have a protective effect [56].

Follow-up experiments interleaved binocular experience with monocular deprivation. Mitchell et al. [78] found that even 0.5 hours of binocular experience with 6.5 hours monocular deprivation preserved moderate spatial acuity in the deprived eye and 2 hours binocular experience with 5 hours monocular experience resulted in normal acuity. Again, they found the inter-ocular correlations were vital, as binocular experience in which artificial strabismus had been induced by prisms resulted in poor recovery of visual acuity. Mitchell et al. [83] found that splitting the binocular experience into discontiguous blocks impeded recovery.

Later experiments explored the neural basis of these changes. Schwarzkopf et al. [17] found normal cortical coverage of both eyes for animals with more than 30 minutes daily binocular experience, whether this was matched with 3.5 or 7 hours of monocular deprivation. Vorobyov et al. [18] examined interocular phase selectivity (a measure of disparity tuning) and found reduced phase selectivity in the partial monocularly reared animals. Mitchell et al. [19] demonstrated that, while partial monocularly reared animals recovered normal levels of spatial acuity in each eye, most had severe deficits in binocular depth perception (unlike normal animals, their depth estimates did not improve when they were allowed to use both eyes). Mitchell et al. [84] showed that animals developed normal spatial acuity provided they received at least 30% binocular experience (regardless of total exposure length) [85]. These results broadly agree that a significant response to the deprived eye recovers with as little as 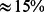 binocular input, and that normal levels of spatial acuity occur with 30% binocular input. However, significant deficits in binocular integration remain even with 30% binocular experience.

**Figure 7:**
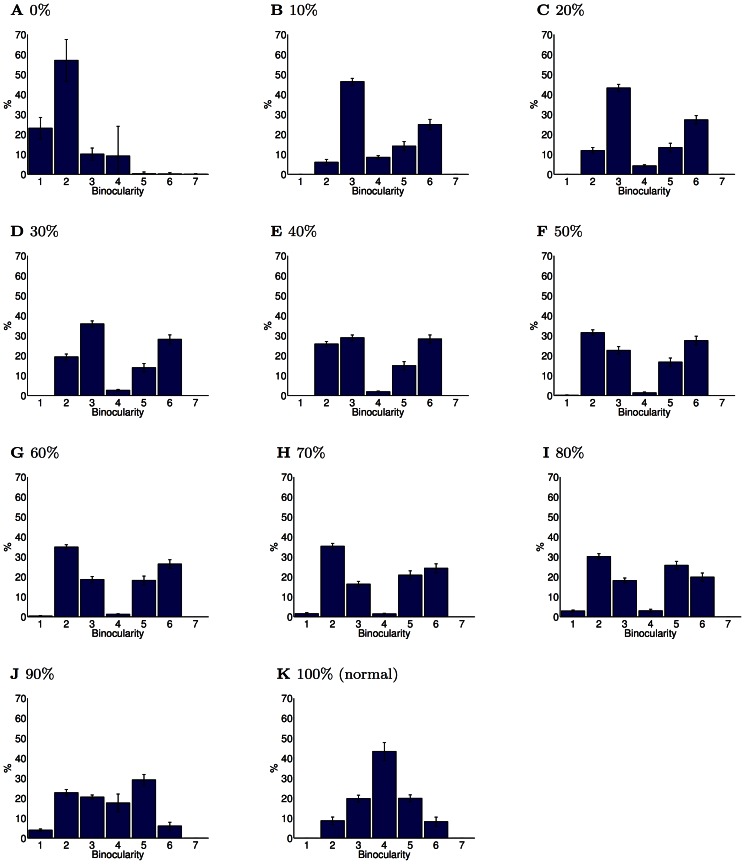
Binocular recovery in partial monocular deprivation (PoE model). This figure shows the ocularity of the receptive fields learned from input with varying fractions of binocular experience (0% corresponds to complete monocular deprivation, 100% to normal binocular experience). (**A**) Complete monocular deprivation resulted in receptive fields unresponsive to the occluded eye. (**B**) Just 10% binocular experience led to a substantial recovery of response to the occluded eye. However, the recovered receptive fields were more monocular than in the normal case. (**C**–**J**) Further increases in the fraction of binocular experience caused a slow recovery of the number of binocular receptive fields. However, even with 90% normal visual experience, neurons were still significantly more monocular than in the normal case. (**K**) Full binocular integration requires normal visual input. Errorbars show the SEM. All the partial monocular rearing conditions had binocularity distributions significantly different from the monocular case (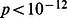, Kolmogorov-Smirnov). Additionally, all cases with partial monocular experience had significantly different binocularity distributions from the normal case (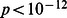, Kolmogorov-Smirnov).

**Figure 8:**
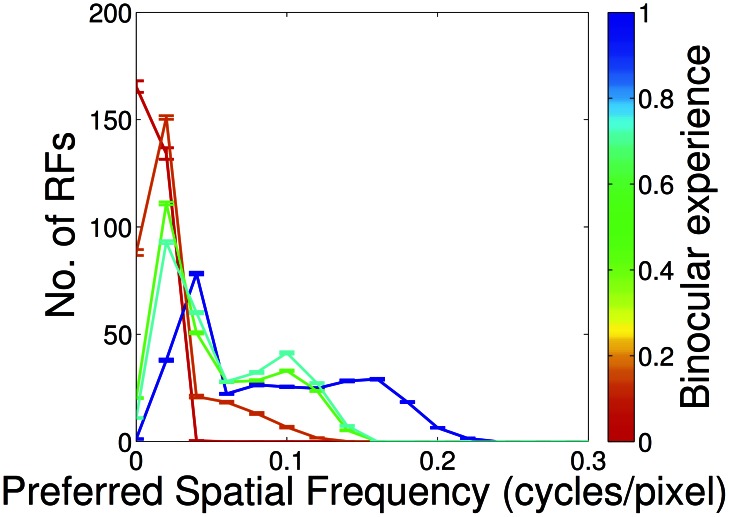
Recovery of spatial acuity in partial monocular deprivation (PoE model). Spatial acuity in the deprived eye recovered rapidly when a small amount of binocular experience was provided. Even 10% binocular experience (orange) was enough to lead to coverage of higher spatial frequencies. However, full binocular experience was required for complete recovery (blue).

We simulated this condition by including a fraction of normal visual input along with the same boxcar filtered input used for monocular rearing. This resulted in significant recovery of deprived eye responses with just 10% binocular input (figure 7**B**), and recovery of equal representation of each eye with 40% binocular input (figure 7**E**) with the PoE model. However, as in the experiments, recovered responses tended to be monocular, with fewer (67% compared with 83% in normal, 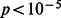 two-sided paired ttest) strongly binocular responses. The deprived eye rapidly recovered responses to the full range of spatial frequencies (figure 8) which corresponds well with the behavioural experiments.

This experimental condition was the only one which showed qualitative differences between the different unsupervised learning models. With the ICA model (Text S1) the recovery from monocular deprivation is much weaker than with the PoE (figure 6) or kmeans clustering (Text S2). Unlike the other models the ICA model is not overcomplete: it has half as many receptive fields to allocate, and may therefore be more susceptible to allocating receptive fields only to the majority input statistics. As we discuss later, there is much evidence that biological V1 is substantially overcomplete.

This recovery appears counterintuitive as it seems the normal input is exerting a disproportionate effect on receptive field development. One explanation may be that, since the sparse coding model strongly penalises representations which are insufficiently sparse, only a small amount of binocular experience is necessary before a significant number of receptive fields are allocated to the deprived eye. As we discuss later, this result suggests that a preferential mechanism for normal input may not be required to explain the recovery observed in partial monocular rearing: rather, it may be a natural consequence of development prior to patterned input.

### Strabismic rearing (figures 1G, 2G, 4G)

Strabismus is of both clinical interest [86], as a condition which affects a significant fraction of the population, and theoretical interest, as it lowers inter-ocular correlations. The effects of convergent or divergent stabismus are similar [87], [88]. Hubel and Wiesel [10] used electrophysiology to show that kittens raised with 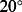 divergent strabismus develop a majority of neurons which are responsive to only a single eye. This finding was reinforced by Shatz, Lindstrom and Wiesel [12] who used histology to demonstrate that strabismic kittens have more bimodal distribution of ocular dominance columns. Chino et al. [89], using animals with strabismus greater than 10°, found no neuron in ocular dominance category 4 (strongly binocular). Unlike other investigators, they also found reduced spatial acuity in the deviating eye and a reduced contrast response. Yinon and Auerbach [87] found that approximately 70% of cells were monocular, and noted an increased number of unresponsive neurons. Similarly, Blakemore [55] measured 76% monocular responses. In a follow-up experiment they observed no strong binocular response when neurons were stimulated optically, and only a small fraction with direct electrophysiological stimulation [74]. Van Sluyters and Levitt [90] used prisms rather than surgical manipulation, which allowed them to create symmetric strabismus. In both divergent and convergent conditions, they found a loss of binocularity with the majority of neurons being assigned ocular dominance categories 1, 2, 6 or 7.

Later results confirmed these findings. Levitt and Van Sluyters [91] found that kittens raised with strabismus during the critical period (2–4 weeks) had cells that were primarily monocular. Grunau [92] measured 80% binocular responses in normal animals and 26% in strabismic. Berman and Murphy [93] also noted a loss of binocularity (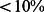 binocular simple cells, compared with 65% for controls), and also observed increased receptive field sizes. Kalil, Spear and Langsetmo [94] found only 7% binocular cells in strabismus. They also reported that animals with divergent strabismus had equal representation of each eye while convergent strabismus resulted in a slight reduction in the representation of the periphery of the deviating eye. Eschweiler and Rauschecker [95] and Schmidt, Singer and Galuske [96] both confirmed that the majority of neurons were monocular. Schmidt Singer and Galuske [96] also found similar orientation preference map characteristics between normal and strabismus (and between maps measured in the deviant and normal eye). Vorobyov et al. [18] also noted a significant decrease in binocular responses compared with control in strabismic animals.

In sum, there is substantial agreement about the effects of strabismus. With 10–20° deviance, whether divergent or convergent, animals develop 80% monocular responses, no strongly binocular response at all, and a reduced number of responsive cells overall. There are mixed reports regarding preferences for the non-deviating eye.

We simulated the effect of strabismus in the models by choosing visual scene patches independently in each eye (focal points were still identical in each). This disrupts inter-ocular correlations, as each eye views different parts of the scene, and led to similar results to those found in the experiments. With the PoE model, only 2% of cells were in ocular dominance category 4, with the majority being in categories 2 and 6 (figure 2**G**). Additionally, there was a significant reduction in the total number of orientation selective cells responding to either eye (figure 3). The full range of orientation preferences continued to be expressed (figure 4**G**).

### Sparse rearing

We have shown above that sparse coding provides an explanation for the RFs that result from several different abnormal rearing conditions. We therefore considered whether there was a novel experiment which could directly address the importance of sparsity in RF envelopment. To do this we exploited that fact that advances in experimental technology have made it possible to rear animals with visual experience which is almost entirely computer-driven (e.g. [97]). This provides nearly unconstrained scope for modification of visual experience after eye-opening.

We sought a stimulus for which sparsity was central, and that we would predict would lead to markedly different receptive fields from normal animals when used as input. It was important that the difference would persist even if some more naturalistic input was additionally provided, for instance from retinal waves present before eye-opening, or from small amounts of natural visual input that cannot be completely controlled in a practical experiment [98]. We also required the stimulus to be statistically stationary in space, so that it would not be necessary to track eye position in presenting the stimulus.

We constructed stimuli by independently sampling each pixel of the patch from a sparse distribution (student-t with 2 degrees of freedom). This is intentionally close to the distribution of coefficients (rather than pixels) in natural scenes, so as to push the model towards learning the Cartesian basis. As a control, we also considered stimuli with a uniform or Gaussian distribution of coefficients. We examined the receptive fields predicted by the model when trained on mixtures of natural scenes and these noise stimuli. All input data were normalized to have the same mean and variance before combination.

**Figure 9:**
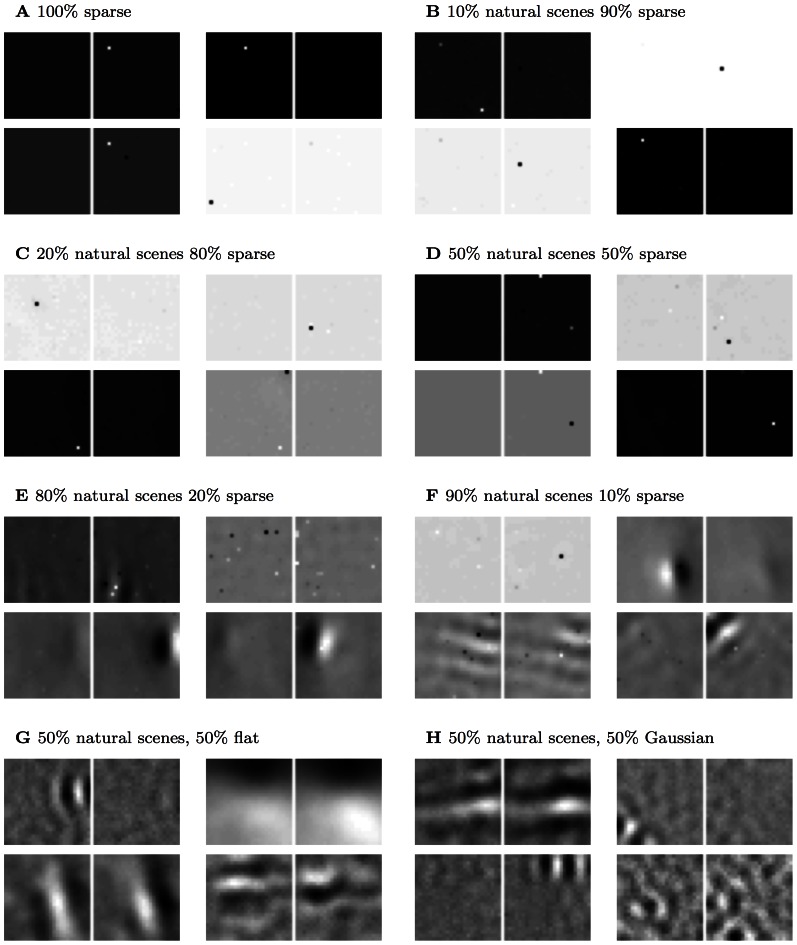
Example receptive fields learned with mixtures of natural scenes and noise (PoE model). We created very sparse noise patterns, with only few pixels with substantial input, and mixed those in various proportions with natural scene input (or other distributions in G, H) for input to unsupervised learning. (**A**) Training the PoE model with 100% sparse noise resulted in highly-localized receptive fields. (**B–F**) Sparse noise continued to have a marked effect on the learned receptive fields even in the presence of natural scene input. With 50% natural input, receptive fields remained strongly localized (D), and even with 90% natural scenes, some pixel localization is still discernable (F). (**G–H**) This result was specific to sparse noise with a coefficient distribution near that of natural scenes. Training the PoE model with a mixture of natural scenes and either uniform white noise (G) or Gaussian (H) mixtures produced weaker perturbations of the receptive fields (cf panel D).

**Figure 10:**
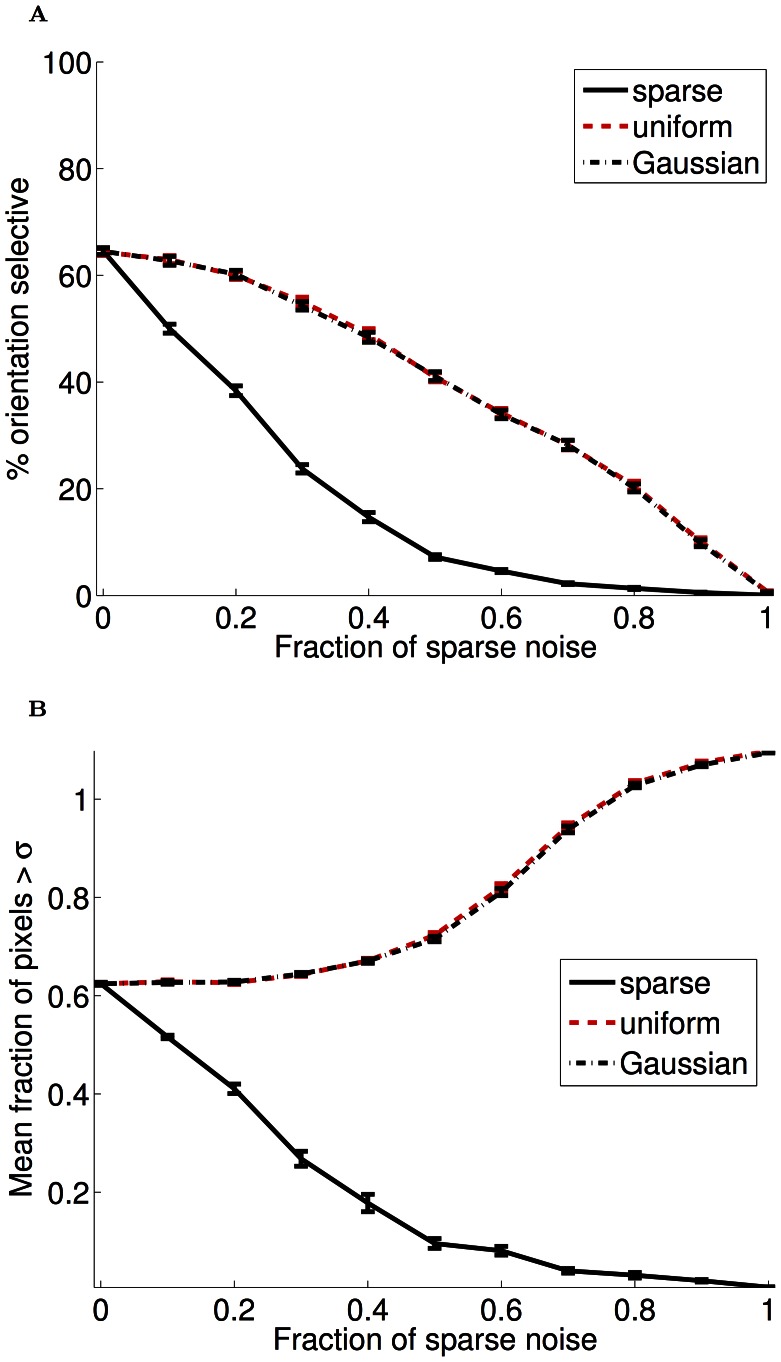
Quantification of receptive field changes with noise (PoE model). (**A**) The fraction of orientation selective responses (defined as circular variance 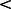
0.6) as a function of the fraction of noise input during training (the remaining input always consisted of natural images). When sparse input constituted greater than 50% of the input, very little orientation selectivity remained. Uniform and Gaussian input also impacted the development of orientation selectivity; however, unlike sparse noise, this impact was more gradual. (**B**) To quantify the extreme localization that is visually apparent in the sparse noise receptive fields, we examined the fractions of weights in each receptive field that were more than 1 standard deviation from the mean of each filter. As with orientation selectivity, when sparse input constituted greater than 50% of the input to the model, most receptive fields were strongly localized to a small number of pixels. The opposite effect occurred with Gaussian and uniform noise, presumably because this input impaired the convergence of the model.

We found that sparse noise provoked a disproportionately strong response in the receptive field development of the models (PoE results in figure 9). Even when trained on a mixture of 50% natural scenes, sparse noise resulted in strongly localized and distinctive receptive fields (figure 10). This effect was particular to sparse noise, as Gaussian or uniform noise had substantially less influence on receptive field development (figure 10).

In this one instance, the k-means clustering model results (text S1) contained some deviation from the results of the other two models. In particular, the development of orientation selectivity was impeded more strongly than in the other models by Gaussian and flat noise, and the mixtures of sparse noise and natural scenes developed a high proportion of low-frequency spatial fields. The exact reasons for these deviations are unclear. However, even with k-means clustering the modification to receptive fields trained with sparse input is large, and robust to the inclusion into the training set of a substantial fraction of natural scene input.

## Discussion

We have shown that sparse coding models can simulate the structure of the V1 simple cell receptive fields that arise when animals are reared with normal and abnormal visual input. Receptive field structure exists prior to eye-opening, and there is ample evidence that important aspects of development are driven by intrinsic factors. However, that dramatic changes in receptive field properties occur that depend on the nature of the input suggests that substantial plasticity remains after eye opening. The account of these changes in our model provides evidence of a causal link between receptive field structure and optimal representations of visual input. This directly answers questions [99] that have been directed at models of purely normal development [26], [27], [30], [32], [37], [100] about the necessity or sufficiency of an impetus towards sparsity. We suggest that sparse coding provides a unifying framework for modelling receptive field changes under a wide variety of rearing conditions.

Understanding the mechanisms by which visual responsivity can be harmed and indeed potentially cured by aberrant or benificent input has important implications. Take, for instance, the case of partial monocular rearing [11], [16]–[19]. Monocularly deprived animals do not develop substantial V1 responses to the occluded eye [13], [14], [63], [64]. Recent experiments have demonstrated the recovery of near-normal visual acuity in animals allowed only a small fraction (1/7) of binocular experience. If this result depended on some intrinsic mechanism for detecting and reacting to this small amount of experience, then it might not generalize. Contrary to this, our findings suggest the enticing possibility that improvements may arise as a natural consequence of developmental optimisation of V1 coding.

Our results regarding the development of receptive fields in abnormal rearing conditions are in agreement with previous results which have examined other modalities and a more limited range of visual rearing conditions. Hsu and Dayan [42] showed that a monocular version of the products of experts model matched the over-representation observed in stripe rearing when trained on stripe filtered input, and a similar result has been found for other algorithms based on sparseness [35]. Saxe et al. [35] also demonstrated that unsupervised learning algorithms could match receptive field changes in abnormal rearing conditions across a range of different sensory modalities.

We used three unsupervised learning methods – independent component analysis [33], product of experts [101] and k-means clustering [102] to acquire sparse codes, thus ensuring that our results did not depend on the specifics of one particular algorithm. As in Saxe et al. [35], we found that all these algorithms learned qualitatively similar sparse codes. One advantage of k-means clustering and the product of experts is that they can readily learn over-complete codes. This is important as V1 is indeed significantly over-complete [23], [28], [42]. Due to the limitations of the experimental data, it is difficult to make any strong claims about which particular unsupervised learning approach provides a better fit. Although the mechanics of these algorithms are not biologically realistic, similar sparse learning algorithms have been implemented in neurobiologically realistic terms [103], [104].

We interpret our results, and those of others based on normal input, as placing the focus on the nature of the efficiency afforded by sparsity [21], [26], [105]–[107]. One notion is that sparse codes may represent a trade-off between the metabolic costs associated with neurons that are firing versus those that are quiescent (and maintaining their membrane potentials; [108]). Of more widespread note is the ability of sparse codes to capture the sort of latent statistical structure in input that can then underpin visual comprehension [105], [109], [110]. The idea is that sensory input arises from the superposition of causes that themselves occur only sparsely. These causes are what it is important to determine. Then, finding a sparse representation of the input in a computationally suitable context (formally, in the recognition component of a pair of recognition and generative models; [110]) can unearth those causes.

The agreement between the experimental results and the output of sparse coding models trained on visual input is perhaps surprising. V1 consists of much more than the feature detectors we have modelled it as here. Real V1 neurons incorporate temporal integration [3], bottom-up and top-down attentional modulation [20], [111]–[113], lateral connections between columns [114]–[116], feature maps [117], [118], and significant feedback from other cortical areas [119]. Additionally, our model does not include the influence of intrinsic activity which is known to be necessary for normal receptive field development [120], wiring constraints [121], and hemispheric asymmetries [16], [79]. These mechanisms may explain the presence of structured receptive fields in dark-reared animals, particularly as spontaneous retinal waves have statistical similarities to natural visual input [122]. However, the success of the model is evidence that, despite many mechanistic differences between the model and V1, sparse codes are good predictors of receptive field changes during the critical period.

Our models favor nurture at the expense of nature. One hint in our results that this favoritism may be too extreme comes from the critical importance of the 10% binocular input in the partial monocular rearing case (and thus the 10% normal input mixed in the stripe and orthogonal rearing conditions). From a technical viewpoint, the primary effect of the normal input is to avoid the collapse of the principal component step which results in the almost complete loss of oriented responses [42], figure 2b. The choice of 10% is somewhat flexible, and Hsu and Dayan [42] found that 75–95% stripe reared input resulted in strong over-representation without a collapse in oriented responses. It could be argued that the 10% is a stand-in for the ineluctable effects of the neural structures established prior to eye-opening, although further work would be required to establish this claim. In the models, the effect of normal input on stripe rearing appears to be more proportionate than in blind rearing; we are not aware of experimental tests of partial stripe rearing for comparison.

Trained with normal input, the models over-represented cardinal orientations. There is evidence that cardinal axes are indeed slightly over-represented in real animals [46], [123]–[127], presumably due to a prevalence of cardinal edges in natural scenes [125], [128], [129]. The degree of over-representation was in reasonably good agreement with a recent study of natural scenes [47]. However, the over-representation in the model may be accentuated because biological retinas are not arranged on a square lattice, unlike most digital representations of images. Such differences in representation are known to exert a small effect on the results of sparse coding models [130].

Modeling binocularity allowed us to observe an effect on coding due to the asymmetry of inter-ocular correlations in visual input that was predicted by Li and Atick [48]. They showed that, because inter-ocular correlation decays more rapidly with horizontal edges than vertical edges, a redundancy reducing code should have an increased number of monocular receptive fields for vertical edges and an increased number of binocular receptive fields for horizontal edges. In our models, despite higher order interactions not considered by Li and Atick, this effect is observed. Hoyer and Hyvärinen [39] have previously examined binocular encoding and reported that learned receptive fields had similar disparity preferences to experiment, but did not report on any relationships between orientation and ocularity. To our knowledge this asymmetry has not been observed in experiment. It is possible that supervised constraints on depth estimation [131] explain why this has not been observed in experiment.

We modelled the six conditions that have been the subject of the most intense investigation. Various other experimental manipulations of visual input have been performed in cats including rearing with random spots [132], exposure to constant speed and direction of motion [133]–[135], astigmatism [136] and opposite rotations of visual input in each eye [137]. We have not attempted to model these results here, either because the studies have not been replicated by other groups or because the effects involved temporal manipulation. In order to constrain the problem size, our models did not include the temporal dimension in receptive field responses, although previous work indicates that this is unlikely to change the results dramatically [37].

However, we did use the models to design a rearing regime that might provide a novel and strong test of the predictive power of sparse coding. According to our models, animals reared with exposure primarily to sparse white noise (similar to that used as a test of the sparse coding algorithm in Olshausen and Field [27]), should develop strongly localized receptive fields. This should be discernable with electrophysiological or optical imaging of receptive fields. Our models predict that the developed receptive fields will be small and mostly non-oriented. This effect is specific to noise with a sparsity near that of natural scenes, and Gaussian noise or distributions with a sparsity much greater than that of natural scenes (such as the spot stimuli examined in Ohshiro, Hussain and Weliky [97]) are predicted to have much less influence, particularly if the animal also receives some naturalistic input [98]. An experimental test of this prediction would provide evidence that sparse coding is a key driver of early receptive field development, or alternatively provide insights into the limits of plasticity during early visual development.

Our models consider only a single cortical area. It would be interesting to look systematically at the effects of the abnormal input statistics on the responses of neurons in higher cortical areas and, concomitantly, on the receptive fields and responses of units in multi-layer, hierarchical [138], [139] unsupervised learning models of those higher areas [35], [140]–[146].

Unsupervised learning can only take us so far in understanding brain function. At some point, brains have goals, seek rewards and avoid punishments. However, transforming high-dimensional input into representations that are more useful is an essential part of artificial forms of machine learning [147], and has offered a critical and realisable metaphor for understanding representations in the brain and the way that they are malleable to changes in the input.

## Methods

### Stereo visual input

We acquired a training set of naturalistic binocular image patches from eighteen stereo images of high-quality, binocular images of natural scenes (from http://home.comcast.net/~toeppen/, this image library was the same as used in Hoyer and Hyvärinen [39]). Each image was photographed using a binocular camera with lenses spaced approximately a pair of human eye's distance apart at varying focal distances. As in Hoyer and Hyvärinen [39], 5 focal points were chosen randomly in each image, the two stereo images were aligned at the focal point and then stereo image patches were acquired in a 300×300 pixel square around the focal point. This has the effect of approximating the view of an observer focussing at 5 different points in the scene. Each patch was 2×25×25 pixels. These patches naturally contain varying degrees of disparity. All images were first converted from color to greyscale. A total of 100,000 training patches were created for each rearing condition.

To model modified rearing conditions, the training input was filtered to match the visual experience of the animals. For stripe and orthogonal rearing the off-axis spatial frequencies were attenuated by using an oriented Gaussian filter [as in 42]. For monocular rearing and alternating monocular rearing the occluded eye's images were convolved with a square kernel with a length of 150 pixels, which removed all but extremely low spatial frequencies. To simulate strabismus, the focal points were chosen independently for each eye. In keeping with previous work, 10% of the training input was unfiltered in the stripe and orthogonal rearing conditions. Hsu and Dayan [42] found that retaining 10% normal input gave a better match with experiment because it reduced the “collapse” of receptive field structure that occurred in the absence of any normal input.

### Learning algorithms

We used three different models for learning sparse codes: product of experts, k-means (which is also known as k-nearest neighbour) and independent component analysis. In all cases, the training data was whitened and dimension reduced using principal component analysis. We retained the first 150 principal components.

We used FastICA [148] to learn independent components and the built-in MATLAB ‘kmeans’ function to learn k-means clustering. Since these algorithms are well-known we do not describe them further here.

The product of experts model [44], [149] models the input distribution as a product of Student-t distributions. Representing each input patch as a column vector 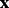, and the ensemble average over all training examples as 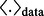, the probability of input 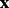 is modelled (with 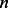 neurons) as:
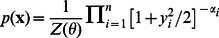
(1)

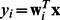
(2)The parameters of this model (encapsulated in the term 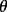) describe the receptive field 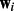 and the sparseness 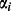 of each neuron 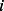. The normalisation constant 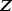 is dependent on 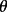. The model is trained by maximising the log-likelihood of the data 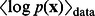 with respect to the model parameters 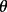. When the number of neurons is equal to the dimensionality of the input there is a closed-form solution for 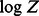 and the model performs independent component analysis [44]. However, when over-completeness is introduced there is no general closed-form solution for the normalisation constant 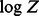. Contrastive divergence [101], which performs gradient descent on a cost function that is within a small constant of the log-likelihood function, was used to fit the model with a learning rate of 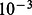 and a batch size of 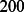. We used an over-completeness factor of 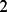, as in [42].

### Characterisation of receptive field properties

Binocularity was quantified using the index described in Hoyer and Hyvärinen [39]:
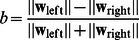
(3)where 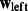 and 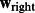 refer to the portions of the receptive field corresponding to each eye and 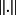 is the 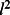 norm. For comparison with experiment we binned the values of 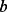 into 7 bins with boundaries at 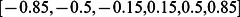 [as in 150]. Bins 1 or 7 correspond to highly monocular responses while values in the middle correspond to binocular responses.

The orientation and spatial frequency preferences for each receptive field was calculated as in Hyvärinen, Hurri and Hoyer [33, ch. 6]. Each eye was treated separately. The response of the receptive field to a quadrature sinusoidal grating over a range of spatial frequencies and orientations was recorded. We examined spatial frequencies between 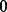 and 0.5 cycles/pixels with spacing of 0.02 cycles/pixels and all orientations with a spacing of 1°. This provided an orientation response curve from which the circular variance [151], which is a measure of the orientation selectivity, could be determined. Circular variance is defined as 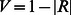 where:
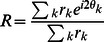
(4)

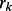
 is the response of the neuron to stimuli of orientation 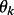. When plotting distributions of orientation preference we only included receptive fields which had a circular variance 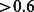, as receptive fields with low circular variance cannot be reliably assigned an orientation.

#### Statistics

In order to test whether the changes observed in the receptive fields under different model conditions were significant, each condition was repeated 25 times with a different pseudo-random seed. The seed affected both the model fitting and the sampling of the training images. Error bars are shown as the standard error of the mean. Except where noted otherwise, tests were performed with a two-sided Kolmogorov-Smirnov test [152]. This test makes only the assumption that the distributions are finite, and tests for differences in both distribution mean and shape.

## Supporting Information

Text S1 A replication of all the figures in the manuscript using the independent components analysis model.
(PDF)

Text S2 A replication of all the figures in the manuscript using the kmeans model.
(PDF)
