# Supplementary material for: Sparse Coding Can Predict Primary Visual Cortex Receptive Field Changes Induced by Abnormal Visual Input
Source: PLoS Comput Biol. 2013 May 9;9(5):e1003005. doi: 10.1371/journal.pcbi.1003005 (PMC3649976; doi:10.1371/journal.pcbi.1003005)
Supplement: Text S1 — A replication of all the figures in the manuscript using the independent components analysis model. (PDF) [file pcbi.1003005.s001.pdf]

Text S1 (ICA)

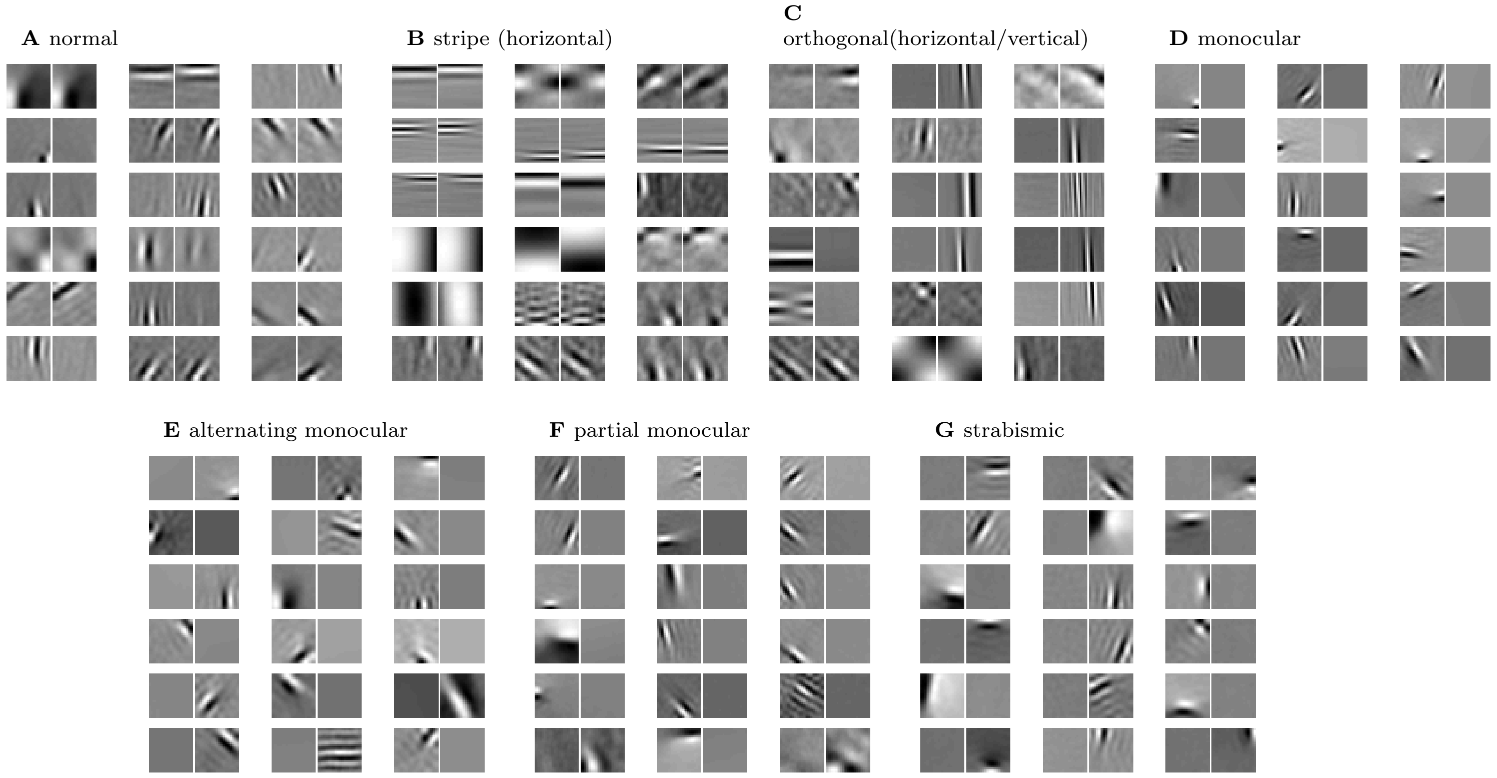

Figure 1. Example receptive fields (ICA model).
Representative examples of the V1 receptive fields over both eyes that result for the ICA model (lower 18 pairs). Subsequent figures quantify the changes in receptive field structure and distribution induced in each rearing condition. See table 1 for a summary of the receptive field changes seen experimentally for each condition. We model rearing with (**A**) normal (unfiltered) visual input, (**B**) stripe rearing, i.e. a single dominant orientation (in this case horizontal), (**C**) orthogonal stripe rearing, i.e. dominant orientations differing by 90 degrees between the two eyes (in this case horizontal and vertical), (**D**) monocular deprivation, i.e. one eye occluded, (**E**) one eye occluded but alternating the eye randomly during training, (**F**) one eye occluded most of the time, and (**G**) artificial strabismus (direction of gaze offset between the two eyes).

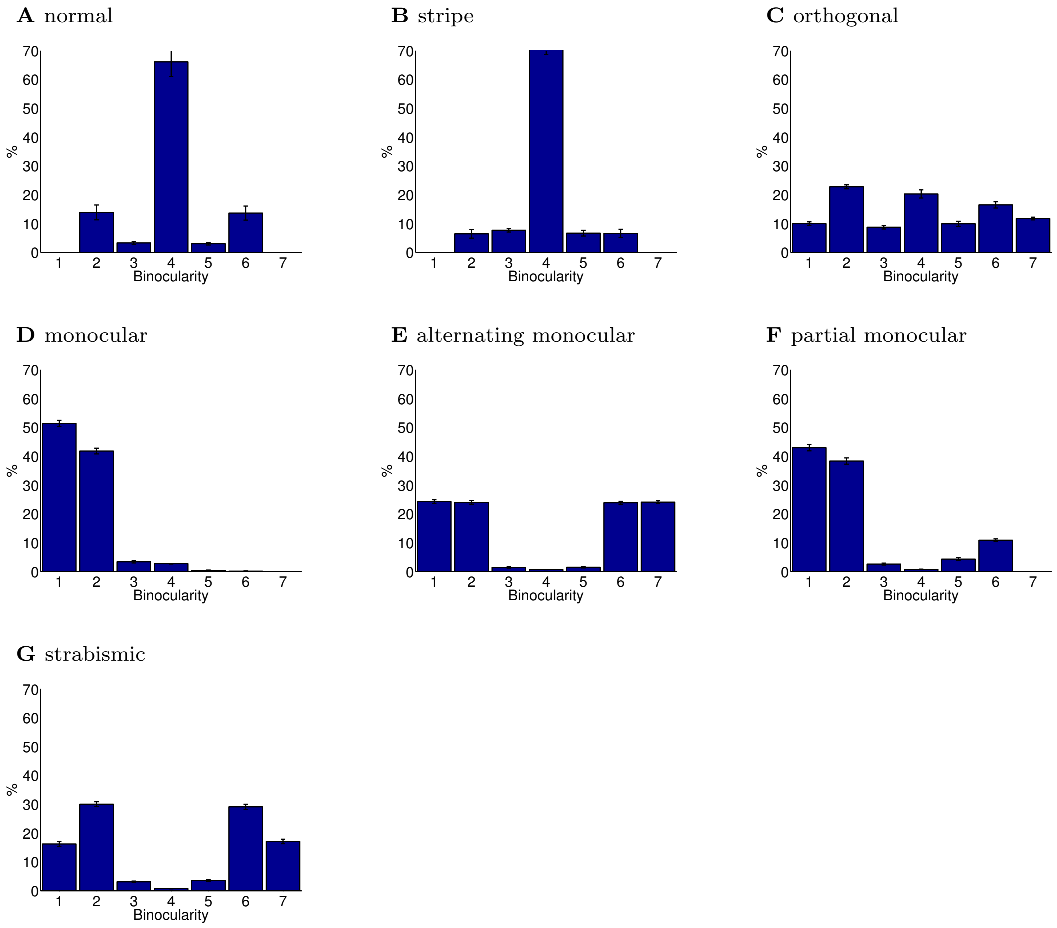

Figure 2. Degree of binocularity for rearing conditions (ICA model).
Binocularity was measured on a 7 point scale as in Shouval et al. [151]. Values 1 and 7 represent completely monocular responses while values in the middle correspond to at least somewhat binocular responses.
(**A**) In the normal rearing condition, neurons had a range of binocular responses, although there were few completely monocular neurons.
(**B**) In the stripe-reared condition, binocularity increased due to higher inter-ocular correlation caused by the reduction in off-axis spatial frequencies. Experiments have also reported varying amounts of increases in binocular responses.
(**C**) In the orthogonal-reared condition, binocularity decreased. Experiments have also reported such a decrease.
(**D**) In the monocular-reared condition, neurons developed responses primarily for the unoccluded eye, which led to strongly monocular responses for this eye. The primary experimental finding in this rearing condition has been the absence of responses to the occluded eye.
(**E**) Alternating monocular rearing removes inter-ocular correlation as each eye is presented with stimuli only when the other eye is occluded. In the ICA model, this led to strongly monocular responses distributed equally between eyes. Experimentally, the primary finding has been a paucity of binocular responses, but equal responses to each eye.
(**F**) Partial monocular rearing resulted in recovery of receptive fields for both eyes, albeit with fewer binocular neurons. Experimentally, a small amount of binocular experience has been found to result in a significant recovery of responses to the occluded eye, but also an increased degree of monocularity.
(**G**) Strabismus decreases inter-ocular correlation, and thus led in the ICA model to increased monocularity. An increase in monocularity is the primary experimental finding of the effects of strabismus.
Errorbars show the SEM. Each condition was repeated $n = 25$ times. The binocularity distribution of all the modified rearing conditions were significantly different from the normal rearing condition ($p < 10^{-8}$, Kolmogorov-Smirnov).

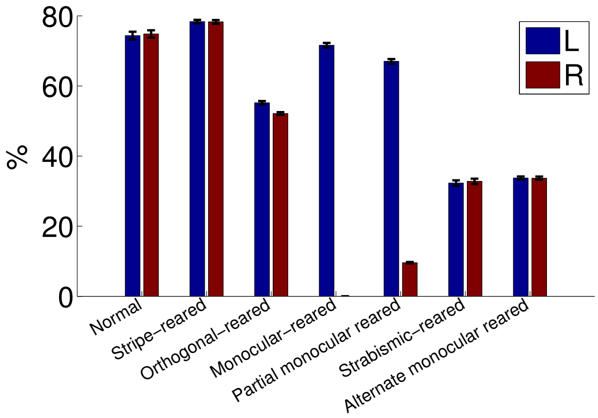

Figure 3. Orientation selectivity across rearing conditions (ICA model).
With the exception of stripe rearing, filtering the visual input resulted in a decrease in the fraction of orientation selective neurons (each eye shown separately). Neurons were considered selective when their circular variance was < 0.6 [3].
Errorbars show the SEM. All modified rearing conditions had a significantly different fraction of orientation selective neurons compared with the normal condition ($p < 0.03$) except stripe rearing ($p = 0.06$, Kolmogorov-Smirnov, each eye tested separately).

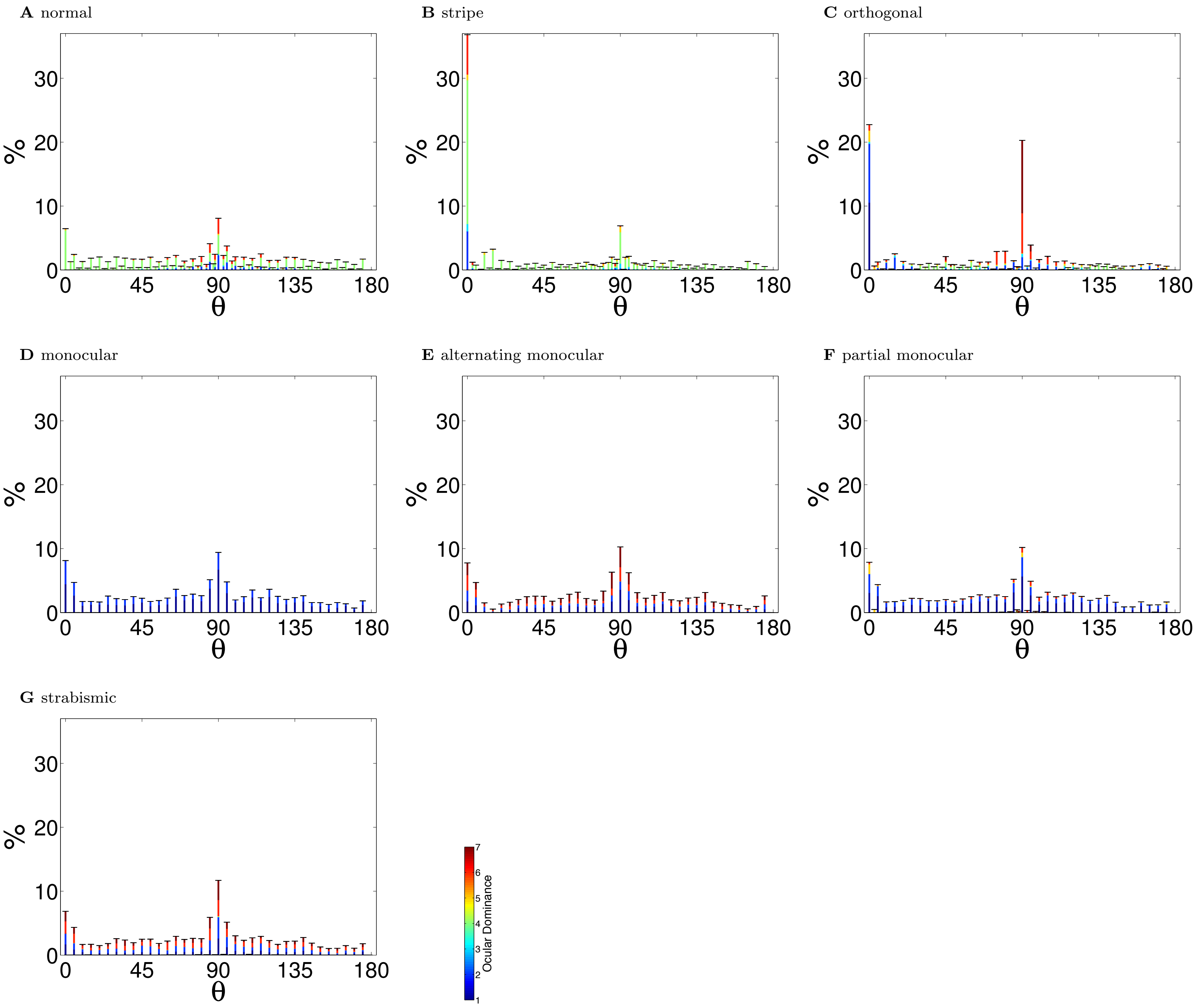
A normal
B stripe
C orthogonal
D monocular
E alternating monocular
F partial monocular
G strabismic
%
θ
0
45
90
135
180
0
10
20
30
Ocular Dominance
1
2
3
4
5
6
7

Figure 4

Figure 4. Orientation preference distributions (ICA model).
Only neurons that had a well defined orientation preference (circular variance < 0.6) in at least one eye were included. The color in the bars indicates the ocular dominance of the responses.
(**A**) In the normal reared condition, there was a large over-representation of vertical orientations (90°), and, to a lesser degree, horizontal orientations (0°). Nonetheless, the full range of orientation preferences developed. This over-representation of the cardinal orientations has been reported in experiment, although not always to the same degree (see the Discussion).
(**B**) In the stripe reared condition, there was a significant over-representation of neurons responding to horizontal lines (0°). These horizontal neurons were also strongly binocular (i.e. mostly green shading). The over-representation of vertical orientations also persisted. The over-representation of the reared orientation is the primary experimental finding in this rearing condition. Cardinal over-representation has not been examined closely in stripe-rearing.
(**C**) In the orthogonally reared condition, there was over-representation of horizontal neurons in the left eye and vertical neurons in the right eye. As found experimentally, these neurons were strongly monocular for the eye that was over-exposed to their preferred orientation.
(**D**) In the monocular reared condition, there was a broad representation of orientation preferences but only for the unoccluded eye. Experimentally, monocular reared animals have normal visual acuity with the non-deprived eye.
(**E**) In the alternating monocular reared case, there is an even distribution of orientation selectivity and strong monocularity. Experimentally, alternate blind reared animals represent all orientations well.
(**F**) In the partial monocular reared condition, there was a recovery of responsivity for both eyes across the full range of orientations. Experimentally, partial monocular reared animals have been demonstrated to have normal visual in each eye (but to suffer from defects in stereo vision).
(**G**) In the strabismic case, there was an increase in monocularity, but with normal orientation coverage. This is in agreement with experiments which have not noted any orientation deficits in strabismic animals.
Errorbars show the SEM. Rearing conditions which modify the orientation distribution had significantly different orientation distributions from the normal case (stripe rearing, orthogonal rearing, $p < 10^-10$, Kolmogorov-Smirnov) while the other conditions did not.

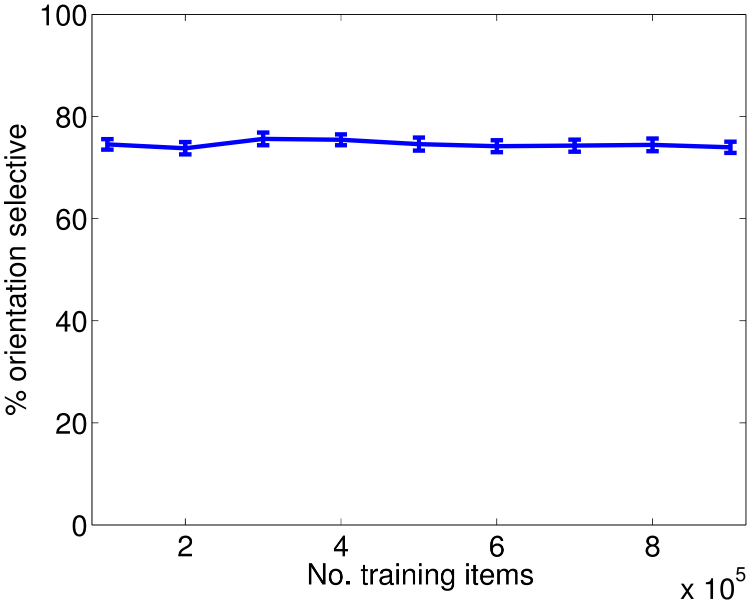

Figure 5. Model is robust to training set size (ICA model).
The ICA model has approximately 200000 degrees of freedom. However, for computational reasons, we trained the model with only 100000 training examples (each training example is projected onto 150 principal components). We therefore examined the effect of increasing the training set size (for the case of normal input). The receptive field orientation selectivity was robust to changes in training set size. There was also no obvious visual change in receptive field structure (data not shown). This demonstrates that the sparsity constraints result in receptive field formation robust to training set size.

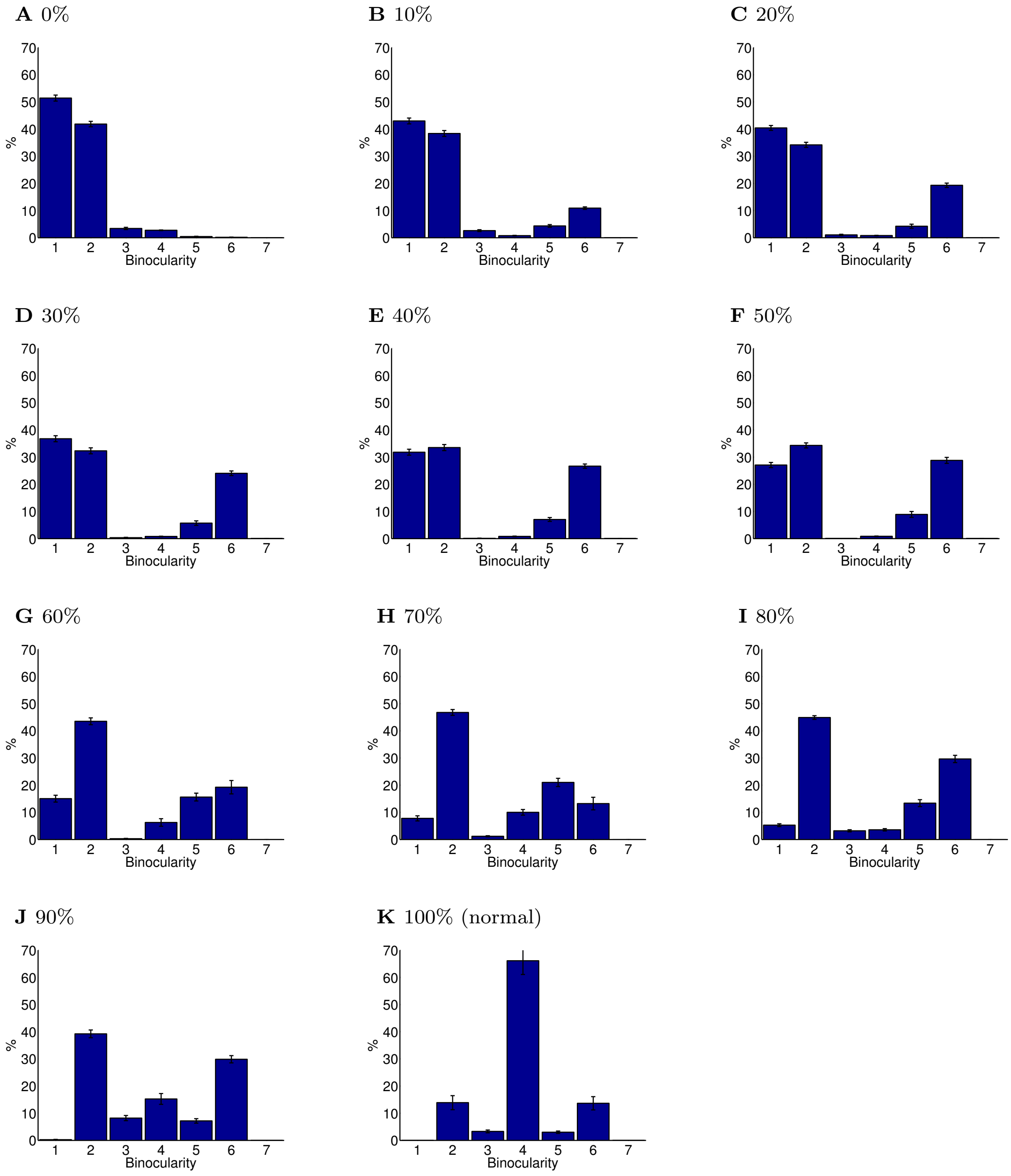

Figure 6. Binocular recovery in partial monocular deprivation (ICA model). This figure shows the ocularity of the receptive fields learned from input with varying fractions of binocular experience (0% corresponds to complete monocular deprivation, 100% to normal binocular experience). (**A**) Complete monocular deprivation resulted in receptive fields unresponsive to the occluded eye. (**B**) Just 10% binocular experience led to a substantial recovery of response to the occluded eye. However, the recovered receptive fields were more monocular than in the normal case. (**C-J**) Further increases in the fraction of binocular experience caused a slow recovery of the number of binocular receptive fields. However, even with 90% normal visual experience, neurons were still significantly more monocular than in the normal case. (**K**) Full binocular integration requires normal visual input.

Errorbars show the SEM. All the partial monocular rearing conditions had binocularity distributions significantly different from the monocular case ($p < 10^{-5}$, Kolmogorov-Smirnov). Additionally, all cases with partial monocular experience had significantly different binocularity distributions from the normal case ($p < 10^{-23}$, Kolmogorov-Smirnov).

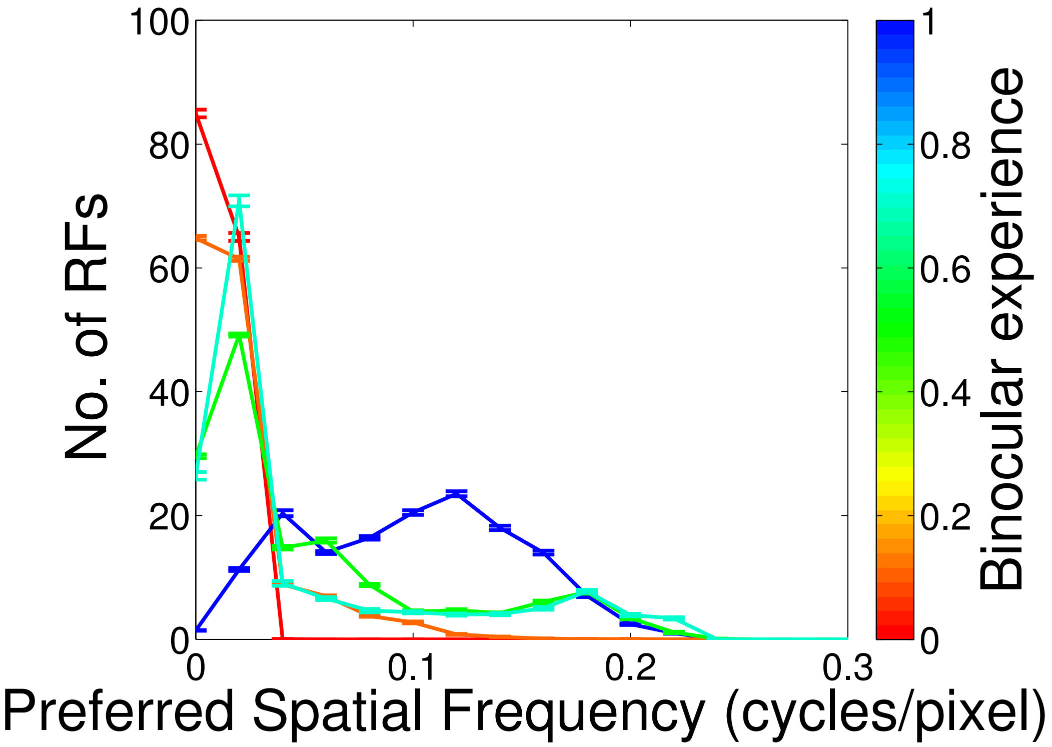

Figure 7. Recovery of spatial acuity in partial monocular deprivation (ICA model). Spatial acuity in the deprived eye recovered rapidly when a small amount of binocular experience was provided. Even 10% binocular experience (orange) was enough to lead to coverage of higher spatial frequencies. However, full binocular experience was required for complete recovery (blue).

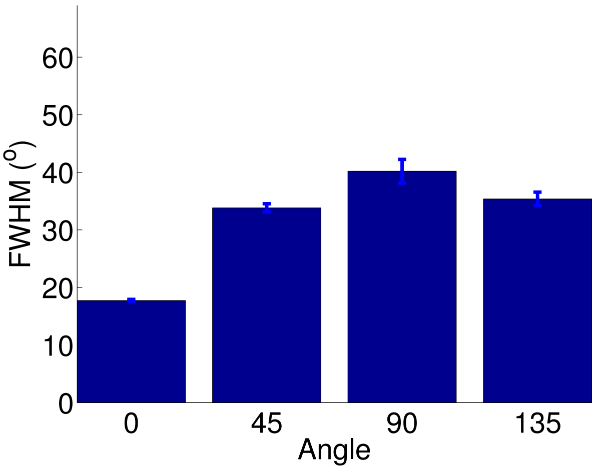

Figure 8. Tuning width in stripe-rearing (ICA model).
In the ICA model, horizontal stripe rearing resulted in significantly sharper orientation tuning for the over-exposed orientation ($p < 10^{-8}$ Kruskal-Wallis). Experimentally, there are conflicting results regarding the tuning of neurons representing the exposed orientation. Tuning width was measured as the full width at half maximum (FWHM) of the tuning curve.

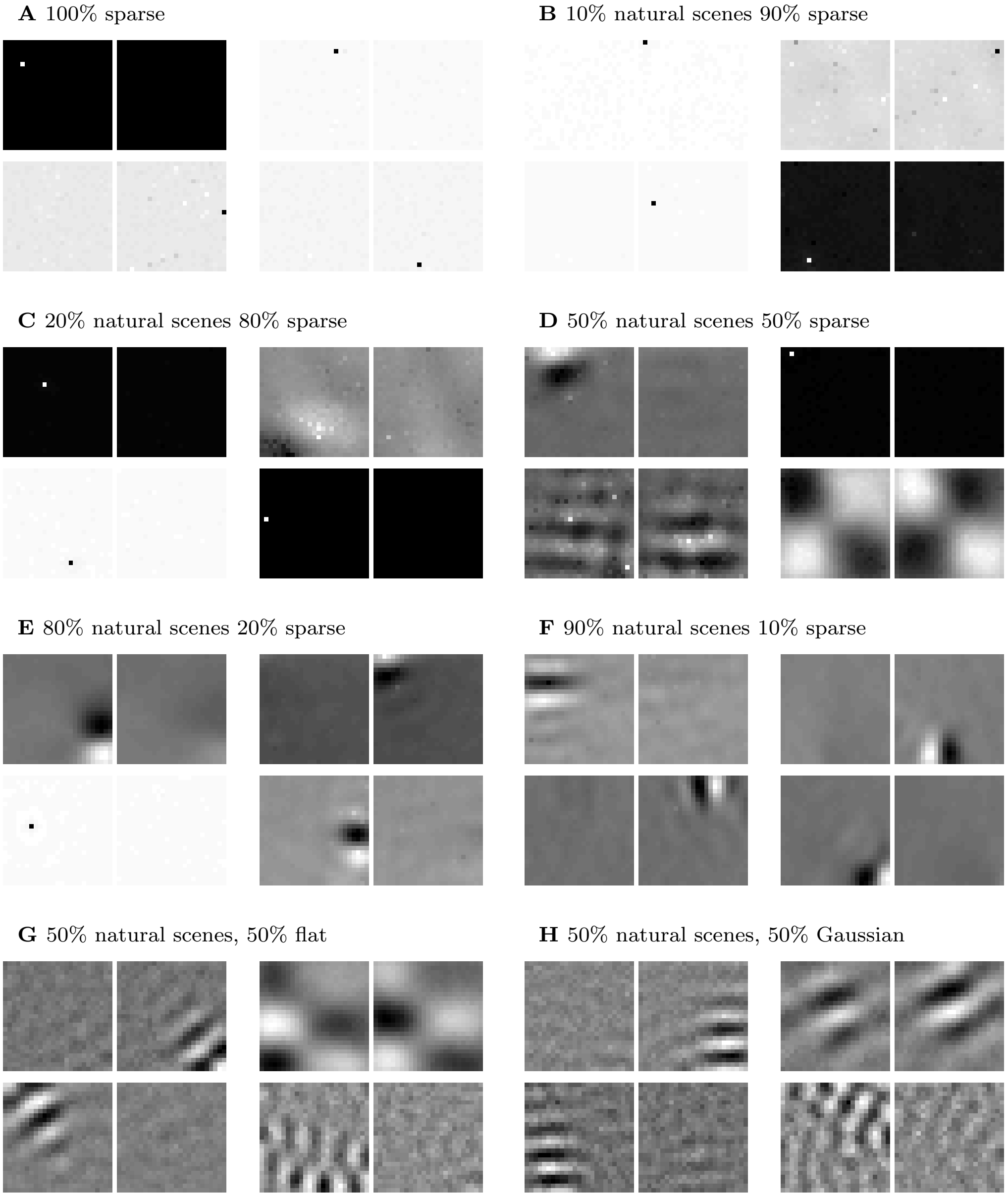

Figure 9. Example receptive fields learned with mixtures of natural scenes and noise (ICA model).
We created very sparse noise patterns, with only few pixels with substantial input, and mixed those in various proportions with natural scene input (or other distributions in G, H) for input to unsupervised learning. **(A)** Training the ICA model with 100% sparse noise resulted in highly-localized receptive fields. (**B-F**) Sparse noise continued to have a marked effect on the learned receptive fields even in the presence of natural scene input. With 50% natural input, receptive fields remained strongly localized (D), and even with 90% natural scenes, some pixel localization is still discernable (F). (**G-H**) This result was specific to sparse noise with a coefficient distribution near that of natural scenes. Training the ICA model with a mixture of natural scenes and either uniform white noise (G) or Gaussian (H) mixtures produced weaker perturbations of the receptive fields (cf panel D).

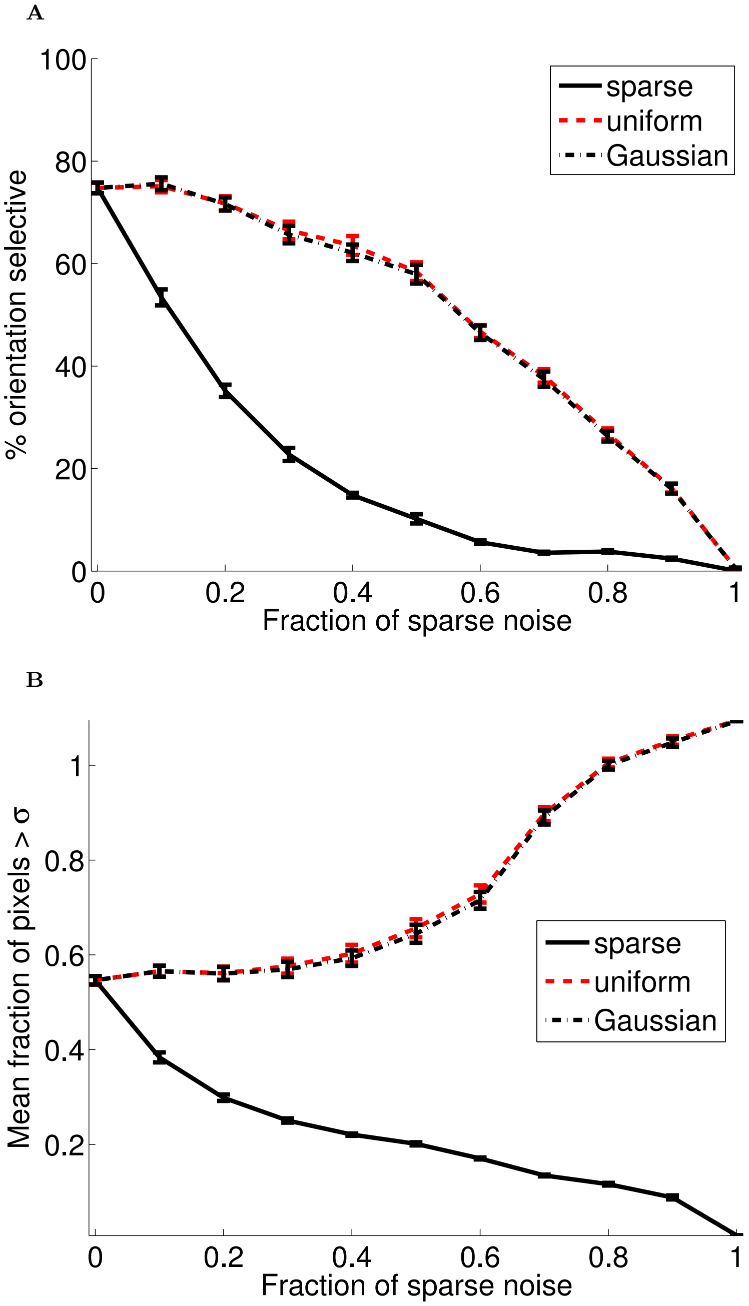

Figure 10. Quantification of receptive field changes with noise (ICA model).
(**A**) The fraction of orientation selective responses (defined as circular variance < 0.6) as a function of the fraction of noise input during training (the remaining input always consisted of natural images). When sparse input constituted greater than 50% of the input, very little orientation selectivity remained. Uniform and Gaussian input also impacted the development of orientation selectivity; however, unlike sparse noise, this impact was more gradual.
(**B**) To quantify the extreme localization that is visually apparent in the sparse noise receptive fields, we examined the fractions of weights in each receptive field that were more than 1 standard deviation from the mean of each filter. As with orientation selectivity, when sparse input constituted greater than 50% of the input to the model, most receptive fields were strongly localized to a small number of pixels. The opposite effect occurred with Gaussian and uniform noise, presumably because this input impaired the convergence of the model.
